# Tumor-derived interleukin-1α and leukemia inhibitory factor promote extramedullary hematopoiesis

**DOI:** 10.1371/journal.pbio.3001746

**Published:** 2023-05-03

**Authors:** Derek A. G. Barisas, Ashraf Ul Kabir, Jun Wu, Karen Krchma, Minseo Kim, Madhav Subramanian, Bernd H. Zinselmeyer, Colin L. Stewart, Kyunghee Choi

**Affiliations:** 1 Department of Pathology and Immunology, Washington University School of Medicine, St. Louis, Missouri, United States of America; 2 Immunology Program, Washington University School of Medicine, St. Louis, Missouri, United States of America; 3 Medical Scientist Training Program, Washington University School of Medicine, St. Louis, Missouri, United States of America; 4 Developmental and Regenerative Biology, A*STAR Skin Research Laboratories, Singapore, Singapore; National Cancer Institute, UNITED STATES

## Abstract

Extramedullary hematopoiesis (EMH) expands hematopoietic capacity outside of the bone marrow in response to inflammatory conditions, including infections and cancer. Because of its inducible nature, EMH offers a unique opportunity to study the interaction between hematopoietic stem and progenitor cells (HSPCs) and their niche. In cancer patients, the spleen frequently serves as an EMH organ and provides myeloid cells that may worsen pathology. Here, we examined the relationship between HSPCs and their splenic niche in EMH in a mouse breast cancer model. We identify tumor produced IL-1α and leukemia inhibitory factor (LIF) acting on splenic HSPCs and splenic niche cells, respectively. IL-1α induced TNFα expression in splenic HSPCs, which then activated splenic niche activity, while LIF induced proliferation of splenic niche cells. IL-1α and LIF display cooperative effects in activating EMH and are both up-regulated in some human cancers. Together, these data expand avenues for developing niche-directed therapies and further exploring EMH accompanying inflammatory pathologies like cancer.

## Abstract 2

This study shows that tumor-produced IL-1α and LIF promote extramedullary hematopoiesis. LIF directly expands splenic niche cells while IL-1α induces TNFα production in hematopoietic stem and progenitor cells to activate splenic niche cells; both expand myeloid-biased splenic hematopoiesis.

## Introduction

Hematopoiesis produces differentiated cell types of the blood and immune systems from hematopoietic stem and progenitor cells (HSPCs). Organismal changes such as disease can modulate the location and cellular output of hematopoiesis [1,2]. Expansion of hematopoiesis outside of the bone marrow (BM), known as extramedullary hematopoiesis (EMH), accompanies pathologic states and occurs mainly within the spleen and liver. Long underappreciated in human disease, EMH is now beginning to be recognized as important component to multiple hematologic and nonhematologic disease [3,4]. The induction of EMH requires mobilization of HSPCs from the BM by chemokines, such as ligands for CXCR2 including CXCL1 and CXCL2 [5–7]. Clinically, EMH presents in a diverse set of solid tumors including breast, lung, renal, colon, gastric, pancreatic, and prostate cancer [8,9]. Of particular interest is EMH in the spleen due to the organ’s role in supplying myeloid cells during multiple injury and disease states and its frequent involvement in cancer patients [8,10,11].

Myeloid-biased differentiation is a response of hematopoiesis to inflammatory signals, including IL-1β, TNFα, and G-CSF [12–14]. Enhanced myelopoiesis, characteristic of EMH, can exacerbate diseases like solid tumors, arthritis, and myocardial infarction by increasing the number of cells that drive pathology [9,15,16]. Clinically, increased myeloid cell production can be measured by an increased ratio of neutrophils (PMNs) to lymphocytes in the peripheral blood (PB). Across multiple tumor types, including breast, colon, pancreatic, and gastric cancer, as well as a systematic review of all cancer types, a high neutrophil-to-lymphocyte ratio in the PB is a poor prognostic factor for survival [17–20]. One potential reason for this observation is the association of chronic inflammatory diseases, like cancer, with the production of immunosuppressive myeloid cells termed myeloid-derived suppressor cells (MDSCs). In cancer, MDSCs, split broadly into granulocytic/polymorphonuclear (PMN-MDSC) and monocytic (M-MDSC) subsets, are postulated to blunt antitumor immunity and prime metastatic niches. Because the presence of MDSCs is beneficial to cancer progression, inhibiting the production or function of MDSCs has drawn attention as a therapeutic modality in cancer [21].

Like other stem cells, hematopoietic stem cells rely on supporting cell types known as the niche [22,23]. Essential to hematopoietic niche function is the production of membrane-bound KIT ligand, a key growth factor for HSPCs [24–26]. Additionally, the niche must produce factors to attract and adhere HSPCs. CXCL12 is a critical chemotactic factor for HSPCs within the BM niche while VCAM-1 is central to adherence through interactions with VLA-4 and other integrins on HSPCs [27,28]. Among hematopoietic niche cell types, perivascular stromal cells play a central role through their production of KIT ligand and CXCL12 [29,30]. Mesenchymal stem cells have been shown to exert niche function in both mice and humans [31,32]. Despite their importance to the niche, demarcating cells as perivascular stromal cells has been tricky. However, several schemas have recognized PDGFRα as an important marker and noted their coexpression of PDGFRβ [33–35]. This PDGFRα+/β+ surface phenotype matches mesenchymal stem cells as identified by single-cell RNA-sequencing (scRNA-seq) of limb muscles [36,37].

Significant advances have been made in delineating the BM niche and HSPC interaction at homeostasis [22,23]. Although perivascular stromal cells have been appreciated as contributing to the splenic niche at homeostasis [38,39], HSPC niches outside of the BM that support EMH are less well understood. Here, we demonstrate the importance of splenic EMH in producing PMNs during a mouse model of breast cancer and identify a novel inflammatory phenotype for HSPCs conducting EMH. We delineate cytokine communication between IL-1α-inflamed, TNFα-expressing splenic HSPCs and activation of their splenic niche. We also show a parallel mode of cytokine communication between tumor cells and the splenic niche through leukemia inhibitory factor (LIF). Both pathways may increase the myelopoietic capacity of the spleen during inflammatory pathologies such as solid tumors.

## Results

### Murine cancer models have expanded splenic hematopoiesis with bias towards myelopoiesis

**Fig 1:**
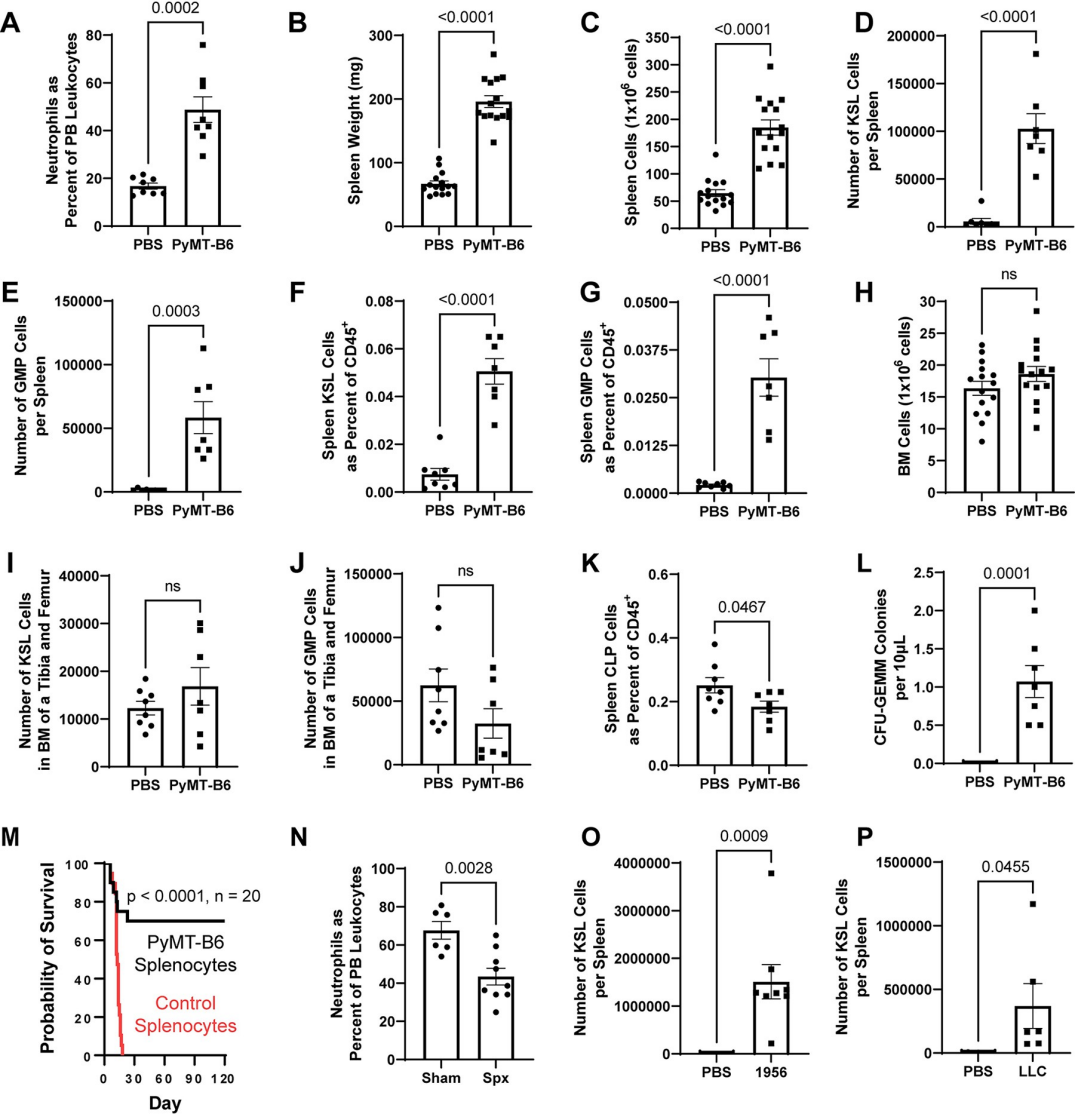
Expansion of splenic hematopoiesis is required for neutrophilia in PyMT-B6. (**A**–**K**) Twenty-one days after injection of 5 × 10^5^ PyMT-B6 tumor cells injected subcutaneously compared to control animals injected with PBS, PMNs in the PB as a percent of total leukocytes (A, *n =* 8), splenic weight (B, *n =* 15), splenic cellularity (C, *n =* 15), KSL cells per spleen (D, *n =* 7–8), GMP cells per spleen (E, *n* = 7–8), KSL cells as a fraction of total splenic CD45^+^ cells (F, *n =* 7–8), GMP cells as a fraction of total splenic CD45^+^ cells (G, *n* = 7–8), BM cellularity per leg (H, *n =* 15), KSL cells per BM of a leg (I, *n* = 7–8), BM GMP cells per BM of a leg (J, *n* = 7–8), CLP cells as a fraction of total splenic CD45^+^ cells (K, *n =* 7–8). (**L**) CFU-GEMM colonies within 10 μL of PB, 21 days of PyMT-B6 tumor compared to PBS-injected controls (*n* = 7–8). (**M**) Survival of 9.5 Gy irradiated mice receiving splenocytes from mice with 21 days of PyMT-B6 tumor or control mice injected with PBS (*n =* 20, significance assigned by Mantel–Cox test). (**N**) PMNs in the PB as a percent of total leukocytes with 21 days of PyMT-B6 tumor following splenectomy or sham surgery (*n =* 7–8). (**O**) KSL cells per spleen 17 days after injection of 2 × 10^6^ 1956 tumor cells injected subcutaneously compared to control animals injected with PBS (*n =* 8). (**P**) KSL cells per spleen 16 days after injection of 5 × 10^5^ LLC tumor cells injected subcutaneously compared to control animals injected with PBS (*n* = 6–7). Processed data for this figure can be found in S1 Data. BM, bone marrow; CLP, common lymphoid progenitor; GMP, granulocyte–monocyte precursor; KSL, Kit^+^/Sca-1^+^/Lineage^−^; LLC, Lewis lung carcinoma; PB, peripheral blood; PBS, phosphate buffered saline; PMN, polymorphonuclear neutrophil; PyMT, polyomavirus middle T antigen.

The MMTV-PyMT mouse is a genetic model of breast cancer where tumors develop in situ due to an oncogene under the control of a promoter expressed primarily in mammary epithelium [40]. Mice with tumors experience neutrophilia and a drastic increase in spleen weight, spleen cellularity, and splenic HSPCs as measured by c-Kit^+^/Sca-1^+^/Lineage^−^ (KSL) and granulocyte–monocyte precursor (GMP) amounts (S1A–S1E Fig). These changes occur with minimal effects to the splenic common lymphoid progenitors (CLPs) (S1F Fig) or the BM compartment (S1G–S1I Fig). To provide more experimental control than the genetic model, we developed a heterotopic tumor transplantation model using a PyMT-B6 cell line derived from tumors of a B6/J syngeneic MMTV-PyMT mouse [41]. PyMT-B6 tumors in female mice aged 8 to 16 weeks, a gender and age range used in further experimentation unless otherwise stated, produced neutrophilia and increased PB and splenic MDSCs of both the PMN-MDSC and M-MDSC subsets in animals 21 days postinjection (Figs 1A and S1J–S1M). Similarly, these mice have increased spleen weight, spleen cellularity, and HSPC (KSL) and GMP amounts, in total and as a percent of CD45^+^ cells (Fig 1B–1G). This effect was not mirrored in the BM compartment or the splenic CLPs (Fig 1H–1K). Additionally, increased CFU-GEMM colony numbers were identified in the PB of PyMT-B6-bearing animals compared to controls, suggesting mobilization of HSPCs outside of the BM (Fig 1L). Together, data from both a genetic and transplantation model identify the spleen as a site of profound HSPC expansion coincident with increased granulocytes and primitive hematopoietic progenitors in the PB.

Enhanced survival of irradiated CD45.2 mice transplanted with CD45.1 splenocytes of PyMT-B6-bearing animals compared to mice receiving nontumor-bearing control splenocytes validated the stem cell function of these splenic HSPCs (Fig 1M). Furthermore, these animals had high levels of donor-derived myeloid and lymphoid lineages in the PB when analyzed at least 1 month after (S1N Fig). Splenectomized animals had reduced PMN percentages in their PB after 21 days of PyMT-B6 tumor compared to sham surgery controls (Fig 1N). To generalize our findings about expanded splenic hematopoiesis to other cancer models, heterotopic models of 1956 sarcoma and Lewis lung carcinoma (LLC) [42–44] were investigated and found to significantly expand HSPC and GMP populations with a variable response to peripheral neutrophilia (Figs 1O, 1P, and S1O–S1R). Together, these data indicate that breast cancer induces expansion of splenic hematopoiesis that is necessary for MDSC-biased neutrophilia and that this expanded capacity is generalizable to other murine cancer models.

### HSPCs conducting EMH express an inflammatory gene profile

**Fig 2:**
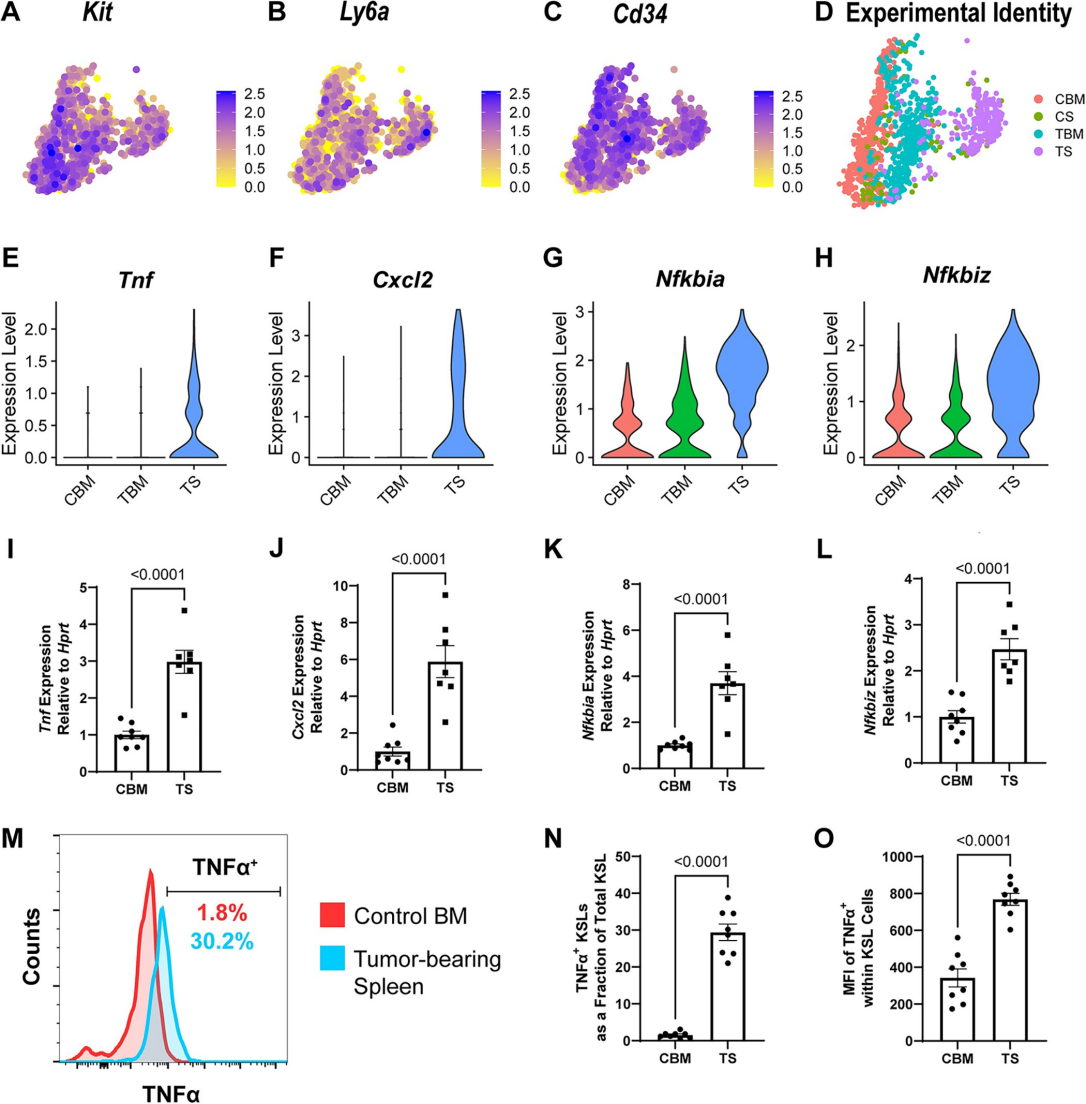
HSPCs from tumor-bearing mice display an inflammatory gene signature. (**A**–**D**) UMAP projection of the HSPC population in scRNA-seq data colored by expression of *Kit* (**A**), *Ly6a* (**B**), *Cd34* (**C**), and by experimental origin (**D**). (**E**–**H**) Violin plot of expression of *Tnf* (**E**), *Cxcl2* (**F**), *Nfkbia* (**G**), and *Nfkbiz* (**H**) in the HSPC population in scRNA-seq data from CBM, TBM, or TS. (**I**–**L**) RT-qPCR expression data of *Tnf* (**I**), *Cxcl2* (**J**), *Nfkbia* (**K**), and *Nfkbiz* (**L**) from Lin^−^/Flk1^−^/CD34^+^ cells from CBM or TS. (**M**) Representative histogram of TNFα expression in KSL cells from CBM or TS. (**N**) Fraction of KSL cells from CBM or TS that are TNFα^+^ (*n =* 8). (**O**) Mean fluorescent intensity of TNFα staining in KSL cells from CBM and TS (*n* = 8). Processed data for this figure can be found in S1 Data. Raw scRNA-seq data are accessible on GEO with the accession number GSE207940. The raw flow cytometry data, gating schema, and staining profile relevant to Fig 2M are deposited on Flow Repository under accession number FR-FCM-Z626. CBM, control BM; CS, control spleen; HSPC, hematopoietic stem and progenitor cell; KSL, Kit^+^/Sca-1^+^/Lineage^−^, RT-qPCR, reverse transcription quantitative PCR; scRNA-seq, single-cell RNA-sequencing; TBM, tumor-bearing BM; TS, tumor-bearing spleen.

To characterize the transcriptional and cell composition changes induced by the PyMT-B6 tumor, we performed scRNA-seq of splenic and BM cells enriched for HSPCs in mice with or without PyMT-B6 tumor. The Lin^−^/c-Kit^+^/Sca-1^+^/CD34^+^ cells representing the hematopoietic progenitor cells (S2A–S2I Fig, clusters 8 and 28; Fig 2A–2C), while rarely present in the control spleen, were well represented in the spleen of the tumor-bearing mice (Fig 2D). The Lin^−^/c-Kit^+^/Sca-1^+^/CD34^+^ cells from the spleen of tumor-bearing animals expressed a unique inflammatory gene signature, including *Tnf*, *Cxcl2*, *Nfkbiz*, *Nfkbia*, compared to control BM (CBM) cells or tumor-bearing BM (TBM) cells (Fig 2E–2H). Intriguingly, some splenic Lin^−^/c-Kit^+^/Sca-1^+^/CD34^+^ cells in a homeostatic mouse displayed a similar inflammatory gene signature, suggesting that even in a homeostatic mouse, tonic inflammatory signals may support very low levels of inflammatory EMH HSPCs (Fig 2D). As such, we chose to focus on the comparison between CBM and HPSCs associated with EMH in the spleen to address questions about the functional differences between homeostatic HSPCs and those associated with pathology and residing in extramedullary sites. The up-regulation of these 4 genes in tumor-bearing spleen (TS) Lin^−^/CD34^+^ HSPC compared to the same population in the homeostatic BM was confirmed by reverse transcription quantitative PCR (RT-qPCR) (Fig 2I–2L). Increased TNFα protein expression was identified within the HSPC fraction of TS relative to CBM by flow cytometry (Fig 2M–2O). Expansion of TNFα expressing splenic KSLs was also identified in the LLC and 1956 tumor models (S2J Fig). Together, these data demonstrate that tumor presence activates an inflammatory gene program within splenic HSPCs to express TNFα.

### PyMT-B6-produced IL-1α acts on HSPCs to express TNFα

**Fig 3:**
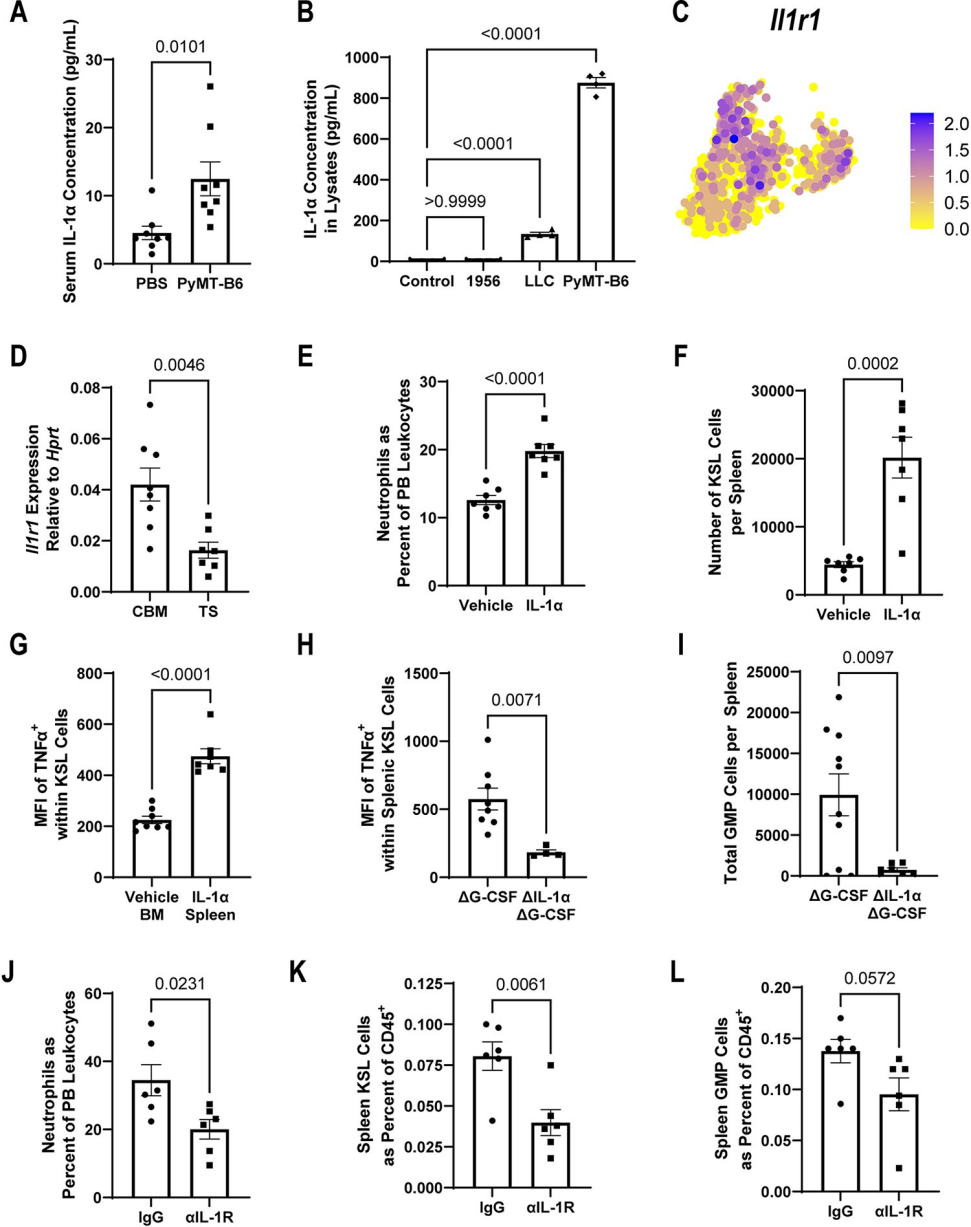
Tumor-derived IL-1α activates TNFα production in splenic HSPCs. (**A**) IL-1α concentration from serum of mice with or without 21 days of PyMT-B6 tumor (*n =* 8). (**B**) IL-1α concentration from 1956, LLC, or PyMT-B6 lysate compared with media control (*n* = 4, significance assigned by one-way ANOVA). (**C**) UMAP projection of HSPCs in scRNA-seq data colored by *Il1r1* expression. (**D**) RT-qPCR expression data of *Il1r1* from Lin^−^/Flk1^−^/CD34^+^ cells from CBM or PyMT-B6 TS. (**E**, **F**) In mice 24 hours after IV injection of 500 ng IL-1α or vehicle, PMNs in the PB as a percent of total leukocytes in mice (**E**, *n =* 7), KSL cells per spleen, (**F**, *n* = 7). (**G**) Average mean fluorescent intensity of TNFα staining in KSL cells from vehicle injected BM or 500 ng IL-1α-injected spleen. (*n* = 7–8). (**H**, **I**) Twenty-eight days after subcutaneous injection of 2.5 × 10^5^ PyMT-B6 ΔG-CSF parental cells or ΔG-CSF ΔIL-1α cells, representative mean fluorescent intensity of TNFα staining in KSL cells (**H**, *n* = 4–8), GMP cells per spleen (**I**, *n* = 7–13). (**J**–**L**) Twenty-one days after injection of 5 × 10^5^ PyMT-B6 tumor cells injected subcutaneously followed by intraperitoneal injections of 200 μg anti-IL-1R antibody or isotype control every third day beginning on day 3 posttumor injection and ending on day 18, PMNs in the PB as a percent of total leukocytes (**J**, *n* = 6, 1 independent experiment), KSL cells as a fraction of total splenic CD45^+^ cells (**K**, *n* = 6, 1 independent experiment), GMP cells as a fraction of total splenic CD45^+^ cells (**L**, *n* = 6, 1 independent experiment). Processed data for this figure can be found in S1 Data. BM, bone marrow; CBM, control BM; GMP, granulocyte–monocyte precursor; HSPC, hematopoietic stem and progenitor cell; IV, intravenous; KSL, Kit^+^/Sca-1^+^/Lineage^−^; LLC, Lewis lung carcinoma; PB, peripheral blood; PyMT, polyomavirus middle T antigen; RT-qPCR, reverse transcription quantitative PCR; scRNA-seq, single-cell RNA-sequencing; TS, tumor-bearing spleen.

TNFα expression in splenic HSPCs of PyMT-B6-bearing mice hints at the presence of tumor-derived upstream mediators. One often reported cytokine subfamily upstream of TNFα is IL-1α and IL-1β [45]; of which, IL-1α, but not IL-1β, is produced during MMTV-PyMT tumor pathology [46]. We confirmed that mice bearing PyMT-B6 tumors have elevated circulating levels of IL-1α (Fig 3A). We also found IL-1α in the PyMT-B6 and LLC cell lysates (Fig 3B). Correspondingly, IL-1 receptor was identified as being constitutively expressed in HSPCs by scRNA-seq and RT-qPCR, although its expression was somewhat lower in tumor-bearing splenic HSPCs compared to CBM HSPCs (Fig 3C and 3D). Single-dose injection of IL-1α into mice was sufficient to induce neutrophilia, increase splenic KSL fraction and total number, and increase TNFα expression in splenic HSPCs compared to CBM HSPCs in 24 hours while not impacting the fraction of splenic GMPs (Figs 3E–3G, S3A and S3B). Reciprocally, deletion of *Il1a* from PyMT-B6 cells led to decreased expression of TNFα in HSPCs and decreased total splenic GMP cells compared to the G-CSF-deleted (Δ*Csf3*) parental PyMT-B6 line (Figs 3H, 3I, S3C and S3D). We deleted IL-1α in the Δ*Csf3*-PyMT-B6 to better understand the role of IL-1α independent of G-CSF, a cytokine which is known to produce myeloid-biased hematopoiesis and EMH that may obscure the effects of other cytokines (S3E–S3G Fig) [47]. Because targeting of IL-1 family cytokines is a clinically utilized therapeutic modality [48], we tested whether blockade of IL-1R signaling by antibody injection impacted neutrophilia and markers of EMH. We found that tumor-bearing mice receiving IL-1R blocking antibody decreased peripheral neutrophilia and the fraction of KSLs in the spleen while trending towards decreased GMP cell fractions in the spleen as well when compared to isotype control-injected animals (Fig 3J–3L). Together, these data indicate that PyMT-B6-derived IL-1α induces a novel inflammatory phenotype in HSPCs associated with tumor-induced EMH that may be targetable therapeutically.

### TNFα induces EMH through splenic niche cells

**Fig 4:**
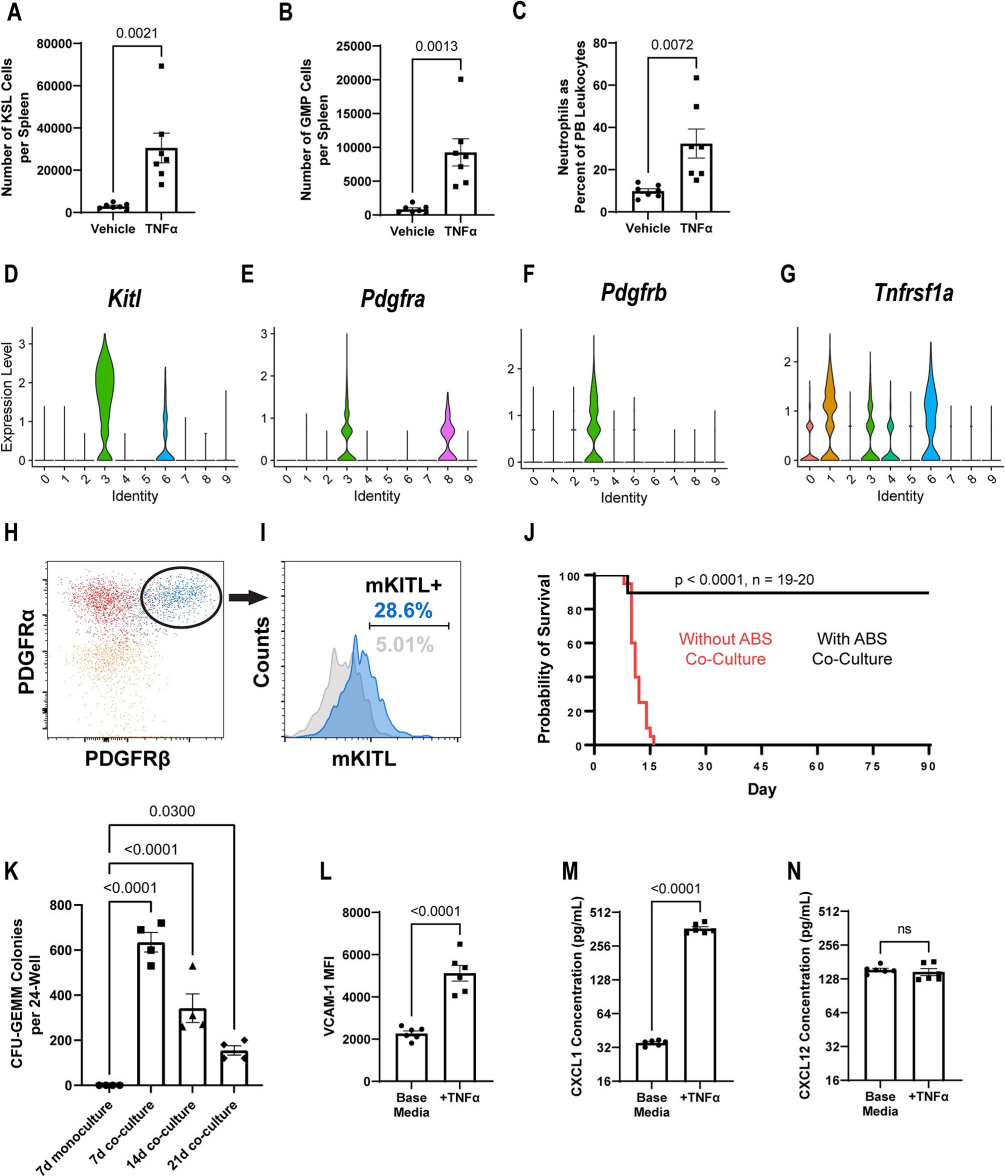
TNFα activates stromal cells in the spleen to induce EMH. (**A**–**C**) In mice 24 hours after IV injection of 2 μg TNFα or vehicle, KSL cells per spleen (**A**, *n =* 7), GMP cells per spleen (**B**, *n* = 7), PMNs in the PB as a percent of total leukocytes (**C**, *n* = 7). (**D**–**G**) Violin plot of expression of *Kitl* (**D**), *Pdgfra* (**E**), *Pdgfrb* (**F**), and *Tnfrsf1a* (**G**) in reanalyzed scRNA-seq data from Tikhonova and colleagues of BM niche cell types (0 –HSC, 1 –endothelium (EC), 2 –proliferating CD45^+^, 3 –ABS cell, 4 –GMP, 5 –CLP, 6 –Sca-1^+^ EC, 7 –B cell progenitor, 8 –osteoblast, 9 –RBC progenitor) [49]. (**H**) Representative dot plot comparing splenic ABS cells stained solely for viability, a PDGFRβ fluorescence minus one sample, and a fully stained sample. (**I**) Representative histogram of membrane KITL expression in splenic ABS cells. (**J**) Survival of 9.5 Gy irradiated mice receiving the cell products of c-Kit^+^Lin^−^ cells grown with or without ABS cell coculture for 7 days (*n =* 19–20, significance assigned by Mantel–Cox test). (**K**) Representative quantification of CFU-GEMM colonies per 24-well of the cell products of c-Kit^+^Lin^−^ cells grown with or without ABS cell coculture for 7 days and with ABS cell coculture for 14 and 21 days. (*n* = 4 replicates per group, significance assigned by one-way ANOVA with multiple comparison tests against the 7d monoculture group). (**L**–**N**) In splenic ABS cells treated for 24 hours with or without 2.5 ng/mL TNFα, representative mean fluorescent intensity of VCAM-1 (**L**, *n* = 6), representative CXCL1 concentration (**M**, *n* = 6), representative CXCL12 concentration (**N**, *n* = 6). Processed data for this figure can be found in S1 Data. The raw flow cytometry data, gating schema, and staining profile relevant to Fig 4H–4I are deposited on Flow Repository under accession number FR-FCM-Z627. BM, bone marrow; CLP, common lymphoid progenitor; EC, endothelial cell; EMH, extramedullary hematopoiesis; GMP, granulocyte–monocyte precursor; HSC, hematopoietic stem cell; IV, intravenous; KSL, Kit+/Sca-1+/Lineage−; PB, peripheral blood; PMN, polymorphonuclear neutrophil; RBC, red blood cell; scRNA-seq, single-cell RNA-sequencing.

Due to the concurrent TNFα production by splenic HSPCs and splenic EMH accompanying PyMT-B6 tumors, we assessed whether TNFα from HSPCs could induce EMH by activating niche cells. Administration of a single dose of TNFα was sufficient to increase HSPC and GMP fractions in the spleen within 24 hours and to produce neutrophilia (Fig 4A–4C). To test whether niche cells respond to TNFα, we needed to identify potential niche cells within the spleen. Reanalysis of a BM niche cell scRNA-seq dataset [49] identified *Pdgfra+/Pdgfrb+* stromal (ABS) cells as being the most strongly KIT ligand-positive cell population and expressing a TNFα receptor (Fig 4D–4G, cluster 3, S4A–S4I Fig). Using a novel method (see Materials and methods section), we cultured ABS cells from the spleen and validated their expression of membrane KIT ligand by flow cytometry (Fig 4H and 4I). To investigate the niche functionality of these ABS cells, 5,000 live BM Lin^−^/c-Kit^+^ (KL) cells, of which around 20% were also Sca-1^+^, were sorted into 24-wells with or without confluent ABS cell cultures. After 7 days of coculture, a large population of small, spherical cells grew on top of the ABS monolayer (S4J and S4K Fig). Upon flow cytometric evaluation, these cocultures contained a population of CD45^+^/Lin^−^/c-Kit^+^/Sca-1^+^ cells (S4L Fig). To test whether the hematopoietic component of these cocultures maintained stem cell capacity, CD45.1 KL cells were sorted into plates and cultured for 7 days with or without ABS stromal before transplanting them into irradiated CD45.2 recipient mice. Compared to mice receiving KL cells cultured without ABS cells, mice that received KL cells cultured on ABS cells had significantly improved survival, indicating the maintenance of repopulating units in vitro (Fig 4J). Analysis of the PB from surviving transplanted mice indicated donor derived hematopoietic cells constituted more than 85% of all CD45^+^ cells after 1 month (S4M Fig). Second, colony-forming unit activity was compared between KL cells grown with or without ABS cells for 7 days. After 7 days, more primitive precursor activity, as measured by CFU-GEMM colony formation, was nearly absent from cells without coculture but preserved in cells grown in coculture (Fig 4K). Additionally, CFU-GEMM colonies were observed until at least 21 days in coculture. Having established genuine HSPC niche activity, we wanted to understand how ABS cells might change phenotypically in response to HSPC cytokines. Following TNFα addition to culture medium, splenic ABS cells increased HSPC-adherent VCAM-1 expression and released the HSPC active chemokine CXCL1 while maintaining baseline CXCL12 release (Fig 4L–4N). Together, these data suggest that TNFα produced by HSPCs in presence of tumor can act on ABS niche cells to increase the capacity of the splenic niche to support hematopoiesis.

### Tumor-derived leukemia inhibitory factor activates splenic EMH

**Fig 5:**
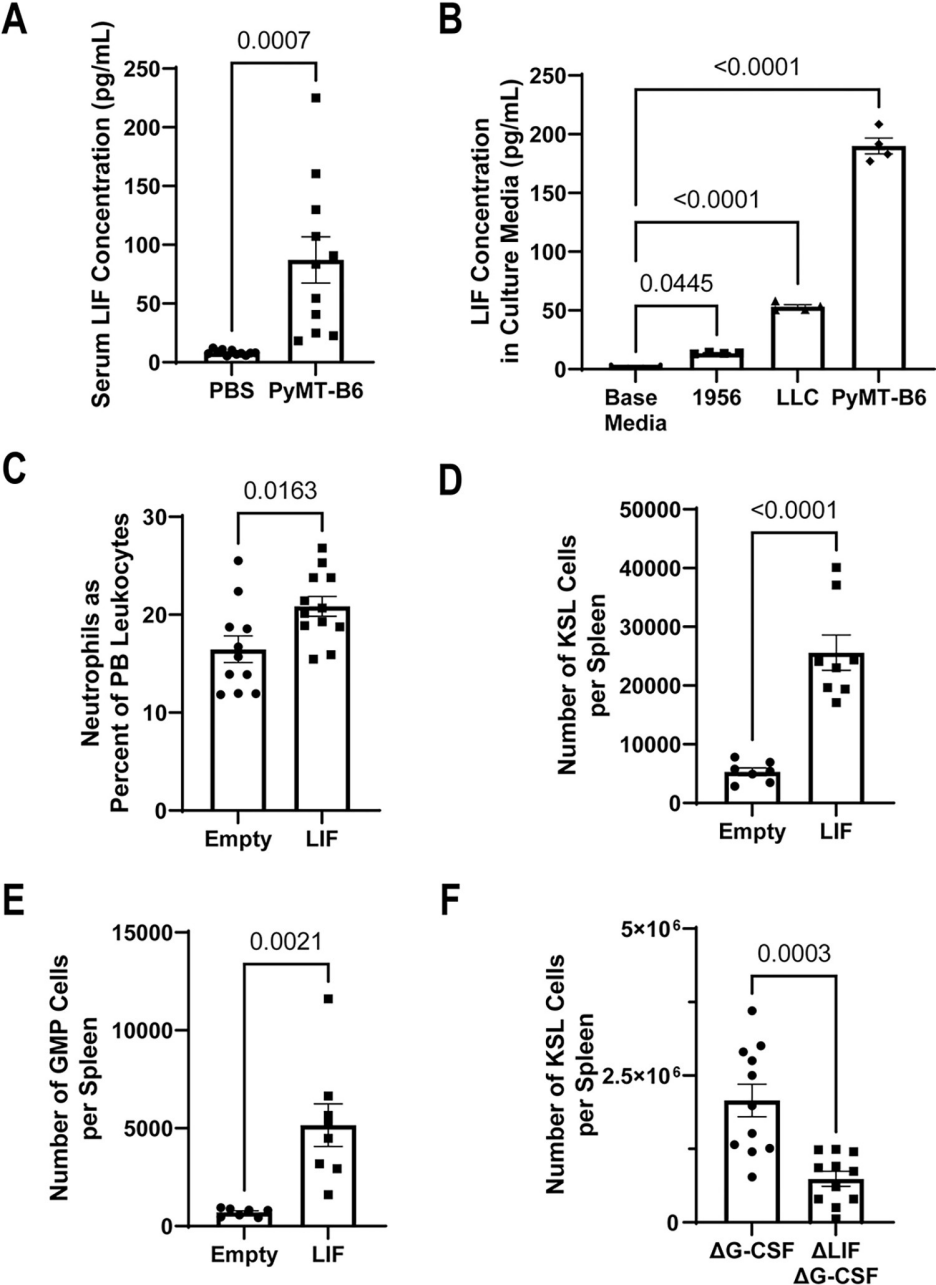
Tumor-produced LIF induces EMH. (**A**) LIF concentration from serum of mice with or without 21 days of PyMT-B6 tumor (*n =* 11). (**B**) LIF concentration from base media, 1956, LLC, or PyMT-B6-conditioned media (*n* = 4, significance assigned by one-way ANOVA). (**C**–**E**) In mice with 10 days of LIF overexpression or empty vector control, fraction of PMNs in the PB as a percent of total leukocytes (**C**, *n* = 11–12), KSL cells per spleen (**D**, *n* = 7–8), GMP cells per spleen (**E**, *n* = 7–8). (**F**) Twenty-eight days after subcutaneous injection of 2.5 × 10^5^ PyMT-B6 ΔG-CSF parental cells or ΔG-CSF ΔLIF cells, KSL cells per spleen (**F**, *n* = 12). Processed data for this figure can be found in S1 Data. EMH, extramedullary hematopoiesis; GMP, granulocyte–monocyte precursor; KSL, Kit^+^/Sca-1^+^/Lineage^−^; LIF, leukemia inhibitory factor; LLC, Lewis lung carcinoma; PB, peripheral blood; PMN, polymorphonuclear neutrophil; PyMT, polyomavirus middle T antigen.

Given the indirect interaction between tumor cells and splenic niche cells through inflamed HSPCs, we were interested in the potential of a direct interaction between tumor and splenic niche cells. Preliminary analysis of a 44-member cytokine array on serum from MMTV-PyMT tumor-bearing animal compared to littermates identified LIF, an IL-6 family member, as being significantly and consistently up-regulated by the presence of tumors (S5A Fig). The presence of LIF in the serum of PyMT-B6-bearing animals and the production of LIF by tumor lines used in this study, 1956, LLC, and PyMT-B6 cells, was independently confirmed (Fig 5A and 5B). Previous work has identified LIF as having an active role in promoting and maintaining hematopoiesis in the spleen [50,51]. We tested whether LIF might have a role in cancer-induced EMH by generating a lentiviral expression vector for murine LIF and injecting mice intravenously to induce systemic LIF overexpression. Compared to empty lentiviral vectors, LIF overexpression induced neutrophilia and a robust expansion of HSPC and GMP cells in the spleen after 10 days (Fig 5C–5E). In contrast, the effect of LIF overexpression on the BM had only minor increases on KSL and GMP frequencies and total numbers (S5B–S5E Fig). Correspondingly, deletion of *Lif* from Δ*Csf3* PyMT-B6 cells lead to decreased levels of splenic HSPCs and GMPs compared to the Δ*Csf3* parental PyMT-B6 line (Figs 5F, S5F and S5G). These data identify LIF as a tumor-secreted factor, which is sufficient to induce myeloid-biased expansion of hematopoiesis predominantly within the spleen.

### Leukemia inhibitory factor induces splenic stromal niche cell proliferation

**Fig 6:**
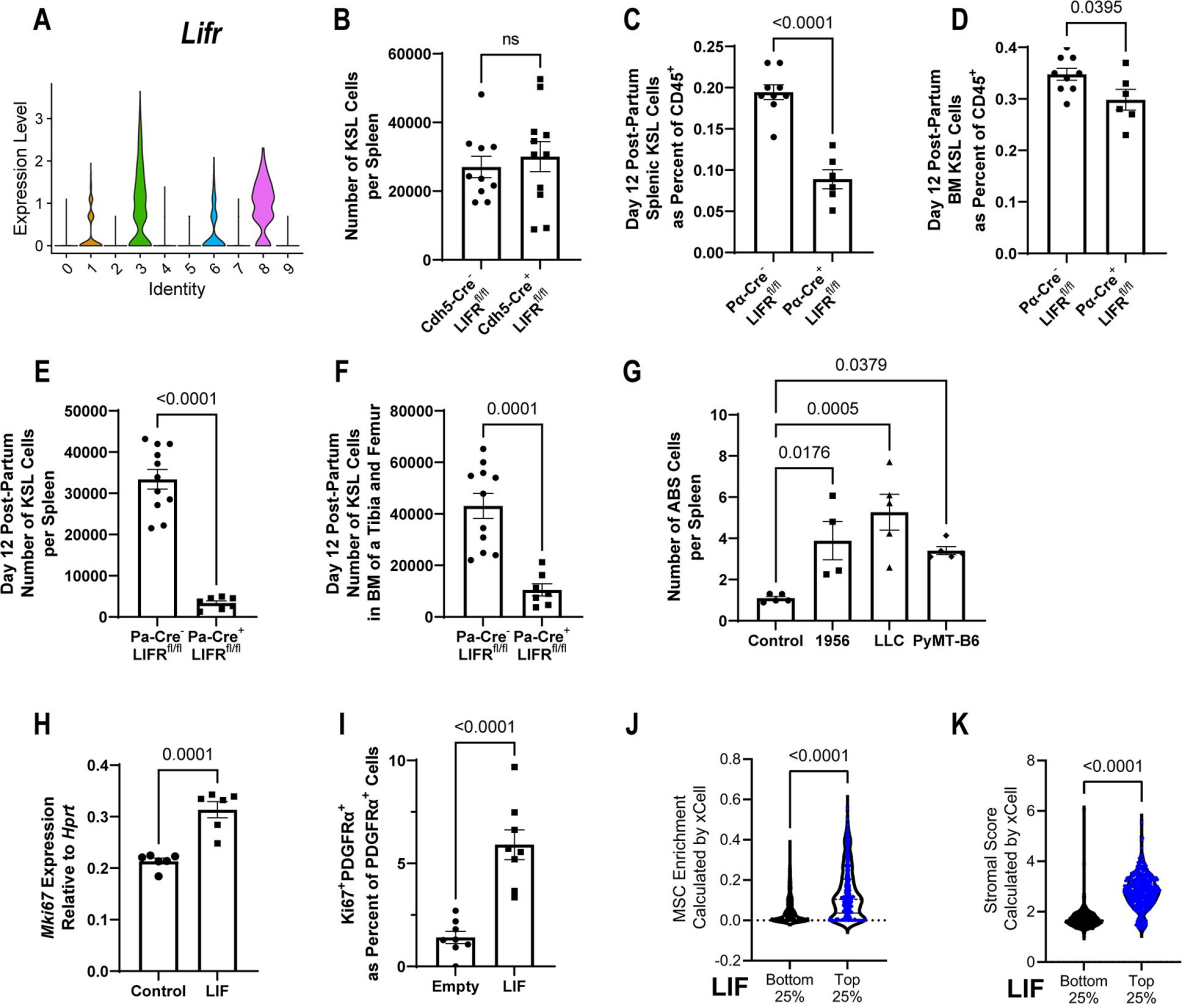
LIF directly expands the splenic niche. (**A**) Violin plot of expression of *Lifr* in reanalyzed scRNA-seq data from Tikhonova and colleagues of BM niche cell types (0 –HSC, 1 –endothelium (EC), 2 –proliferating CD45^+^, 3 –ABS cell, 4 –GMP, 5 –CLP, 6 –Sca-1^+^ EC, 7 –B cell progenitor, 8 –osteoblast, 9 –RBC progenitor). (**B**) In LIFR^flox^ mice with LIF overexpression and Cdh-Cre^+^ or Cdh5-Cre^−^, KSL cells per spleen (*n =* 10–11, contains male mice). (**C**–**F**) In day 12 postpartum LIFR^flox^ mice with PDGFRα-Cre^+^ or PDGFRα-Cre^−^ littermates, KSL cells as a fraction of total splenic CD45^+^ cells (**C**, *n* = 6–9, contains male mice), KSL cells as a fraction of total BM CD45^+^ cells (**D**, *n* = 6–9, contains male mice), KSL cells per spleen (**E**, *n* = 6–9, contains male mice), KSL cells per leg (**F**, *n* = 6–9, contains male mice). (**G**) Twenty-one days after injection of 2 × 10^6^ 1956 tumor cells, 5 × 10^5^ LCC tumor cells, or 5 × 10^5^ PyMT-B6 tumor cells, injected subcutaneously compared to control animals injected with PBS, total ABS cells per spleen (*n* = 4, 1 independent experiment, significance assigned by one-way ANOVA). (**H**) Representative RT-qPCR expression data of *Mki67* from splenic ABS cells treated for 72 hours with 20 ng/mL LIF (*n* = 6). (**I**) Fraction of splenic PDGFRα+ cell that are Ki67+ by immunofluorescence with 7 days of LIF overexpression or empty vector lentivirus control (*n* = 8). (**J**, **K**) Enrichment of MSCs (**J**) and stromal scoring (**K**) as calculated by xCell from RNA-seq data of human tumors split by top and bottom quartile of LIF expression (*n* = 416–417). Processed data for this figure can be found in S1 Data. BM, bone marrow; CLP, common lymphoid progenitor; EC, endothelial cell; GMP, granulocyte–monocyte precursor; HSC, hematopoietic stem cell; KSL, Kit^+^/Sca-1^+^/Lineage^−^; LLC, Lewis lung carcinoma; LIF, leukemia inhibitory factor; MSC, mesenchymal stromal cell; PBS, phosphate buffered saline; PyMT, polyomavirus middle T antigen; RBC, red blood cell; RT-qPCR, reverse transcription quantitative PCR; scRNA-seq, single-cell RNA-sequencing.

Having identified the capability of LIF to expand splenic hematopoietic capacity, we sought to define a cellular mechanism for its effect. Reexamination of niche scRNA-seq data [49] identified both *Kitl* expressing clusters, *Cdh5+/Ly6a*+ endothelial and ABS cells, as expressing LIF receptor (LIFR) (Fig 6A, cluster 6 versus 3, respectively). By inducing LIF overexpression by lentivirus in Cdh5-Cre^+^; Lifr^fl/fl^ mice and littermate controls, we could exclude endothelial cell contribution to LIF-induced EMH (Fig 6B). We also generated mice with LIFR deletion within the PDGFRα^+^ population to assess the involvement of splenic ABS cells in LIF response. Pdgfra-Cre^+^; Lifr^fl/fl^ mice were born at expected frequencies but died before weaning due to a failure to thrive, a similar but less severe phenotype than the constitutive knockout mouse (S6A and S6B Fig) [52]. Despite the lethality at around the weaning, conditional knockouts are still alive at days 12 postpartum, a time point when the spleen still shows active hematopoiesis [53]. We found that Pdgfra-Cre^+^; Lifr^fl/fl^ mice had reduced HSPCs predominantly within the spleen compared to the BM and littermate controls at this time (Fig 6C–6F). This suggests that the LIF-LIFR axis in PDGFRα^+^ cells is indispensable for maintenance of hematopoiesis specifically within the spleen.

Examination of total ABS cell numbers per spleen in the 1956, LLC, and PyMT-B6 tumor models found increases in ABS cells across all 3 models when compared to control spleens (Fig 6G). Previous studies have shown that LIF induces proliferation of PDGFRα+ oligodendrocyte precursor cells and osteoblast precursors [54,55]. We added LIF to splenic ABS cultures and also found increased markers of proliferation (Fig 6H). To confirm this finding in vivo, we quantified the fraction of Ki67^+^ nuclei of PDGFRα^+^ cells in the spleen with or without lentiviral LIF overexpression using immunofluorescence and found an increase in Ki67^+^ PDGFRα^+^ cells with LIF overexpression (Fig 6I and S6C). Additionally, we found the close association of PDGFRα^+^ cells with Kit^+^ progenitors in the spleen after LIF overexpression by confocal imaging (S6D Fig).

Our data suggest that LIF produced by tumor expands distal stromal components in mouse models. To investigate whether LIF expression in human cancer correlates with local stromal populations, we analyzed RNA-sequencing data from public human tumor datasets [56]. Consistent with our mouse data, tumors in the highest quartile of LIF expression had significantly higher amounts of MSCs, fibroblasts, and stromal scores compared to the lowest quartile, with only a modest increase in the endothelial fraction between the two groups (Figs 6J, 6K and S6E). Together, these data suggest that ABS cells form an expandable niche in the spleen in direct response to tumor-derived LIF and that this cancer-stromal interaction may operate in human tumors as well.

### IL-1α and LIF have a cooperative myelopoietic response in mice and are coexpressed in human cancers

**Fig 7:**
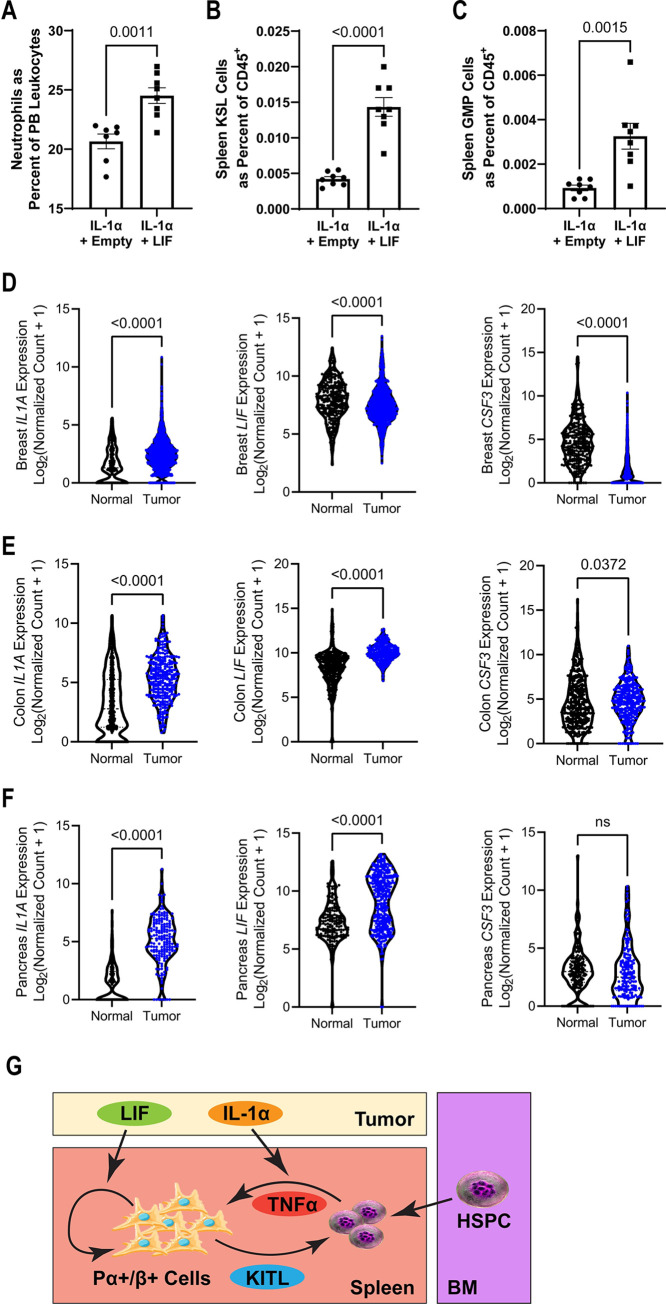
**Human tumors coexpress LIF and IL-1α, which synergize in mouse models to potentiate EMH.** (**A**–**C**) In mice with 10 days of LIF overexpression lentivirus or empty vector control lentivirus and 24 hours after treatment with 200 ng IL-1α IV, PMNs in the PB as a percent of total leukocytes (**A**, *n =* 7–8), KSL cells as a fraction of total splenic CD45^+^ cells (**B**, *n* = 7–8), GMP cells as a fraction of total splenic CD45^+^ cells (**C**, *n* = 7–8). (**D**–**F**) RNA-seq expression of *IL1A*, *LIF*, and *CSF3* expression in tumor compared to normal tissue for breast (**D**, 292–1,099), colonic (**E**, *n* = 288–349), and pancreatic (**F**, *n* = 171–178). (**G**) Our proposed model of parallel mechanisms for tumor-associated EMH mediated in part by indirect inflammatory changes to HSPCs by tumor-derived IL-1α through local HSPC TNFα expression and direct proliferative effects on splenic ABS cells from tumor-derived LIF. Processed data for this figure can be found in S1 Data. EMH, extramedullary hematopoiesis; GMP, granulocyte–monocyte precursor; HSPC, hematopoietic stem and progenitor cell; IV, intravenous; KSL, Kit^+^/Sca-1^+^/Lineage^−^; LIF, leukemia inhibitory factor; PB, peripheral blood; PMN, polymorphonuclear neutrophil; RNA-seq, RNA-sequencing.

Due to their independent mechanisms in activating the splenic niche, we determined if the interaction of IL-1α and LIF would increase myelopoietic output. To this end, we first injected mice with lentiviral constructs that were either empty or expressed LIF, followed by IL-1α. Mice that had received both LIF and IL-1α had increased peripheral PMNs and splenic HSPCs and GMPs compared to IL-1α alone (Fig 7A–7C). These data suggest that LIF exerts a functional impact on hematopoietic capacity that can potentiate the myelopoietic impact of IL-1α.

The PyMT-B6 mouse breast cancer line expresses IL-1α and LIF in addition to G-CSF. To examine whether the combination of IL-1α and LIF in the absence of G-CSF is relevant to human disease, we reexamined human tumor RNA-sequencing datasets from TCGA and other sources. We found that human breast cancer does not have the same cytokine profile as PyMT-B6 as it only overexpresses IL-1α but not LIF or G-CSF (Fig 7D), while human colon cancer was the only cancer to overexpress all 3 cytokines (Fig 7E). Importantly, human pancreatic, stomach, brain, and bile duct cancer all have overexpression of both IL-1α and LIF relative to normal tissue while having only minor changes in G-CSF (Figs 7F and S7A–S7G). These data suggest that the co-occurrence of IL-1α and LIF is likely clinically relevant for a diverse set of human cancers. Collectively, our data illuminate a novel, potential mechanism by which murine and human cancers may generate an immunosuppressive, myeloid-biased immune environment through expanded HSPC niche capacity in the spleen, initiated in part by tumor-derived cytokine factors (Fig 7G).

## Discussion

EMH can be viewed as a process undertaken to meet the immense demand for myeloid cells during pathology that exceeds the capacity for existing BM progenitors, making EMH a mechanism of emergency hematopoiesis. While EMH has been shown in a wide range of inflammatory conditions and diseases [3,4,8], the mechanisms regulating EMH have not been clearly elucidated. Here, we show the spleen as a critical site of EMH during solid tumor pathology that drives increases in PB neutrophilia. Consistent with this is recasting the spleen as a primary lymphoid organ involved in sensing systemic inflammation and activating by expanding total hematopoietic capacity, often with a myeloid bias. This framing of splenic function is concordant with data demonstrating an origin for myeloid cells within the spleen during various inflammatory and non-inflammatory pathologic states in both humans and mice [10,11]. Previous work in a hepatocellular carcinoma mouse model has demonstrated that the absence of the spleen is sufficient to positively impact immune checkpoint blockade therapy [9]. Additional work has suggested that absence of the spleen leads to significantly fewer tumors developing in an inducible model of lung cancer [11]. We also show the reduction in the magnitude of tumor-induced neutrophil bias in the periphery following splenectomy. Intriguingly, our study finds less profound alterations to the BM compartment when compared to other investigations of solid tumor induced effects on hematopoiesis. In particular, Casbon and colleagues found a trend towards increased BM cellularity and progenitor numbers using the MMTV-driven PyMT transgenic breast cancer model [57]. Potentially, these discrepancies are the result of several differences between our experimentation and theirs including mouse genotypes, a transgenic versus tumor transplantation model, and a longer timeframe in the transgenic model. Additionally, we also speculate that increases to BM HSPCs seen in the previously mentioned study and others may contribute to increased splenic HSPCs through migration as observed in our model. These subtle differences and their impact on stem cell phenotypes highlight the nuance and limited understanding still present in the field of hematopoietic modulation by solid tumors.

While tumor manipulation of local immune cells within the tumor microenvironment has received significant attention, how tumor cells manage the immune system distally is less well understood. In this paper, we demonstrate that profound expansion of hematopoiesis into the spleen occurs with breast cancer. We identify 2 cytokines produced by tumor cells that have distinct but overlapping interactions with splenic HSPCs and stromal cells to expand the size and functional capacity of the splenic niche to accommodate increased myelopoiesis. We present novel findings that support this conclusion. First, splenic HSPCs accompanying tumor presence express a gene profile characterized by TNFα. Second, IL-1α released by the tumor cells acts distally to induce TNFα expression in HSPCs. In this scheme, it is also possible that IL-1α can be produced by nontumor cells. Third, tumors may indirectly activate splenic niche capacity in PDGFRα+/β+ stromal cells through local TNFα produced by inflammatory HSPCs. Although our paper cannot rule out the importance of TNFα produced by non-HSPCs acting on the niche, the long-recognized function of close association between HSPCs and their niche and the important functional role for transmembrane and soluble TNFα lead us to conjecture that this local bidirectional circuit is biologically important [23,58]. Fourth, tumors directly expand the splenic niche through LIF by inducing proliferation in splenic PDGFRα+/β+ stromal cell populations. Moreover, LIFR deletion in PDGFRα+ cells significantly reduced hematopoietic capacity within the spleen. These data extend the role of the ABS cell type by centering it as the activatable niche cell within the spleen. Importantly, identifying LIF as expanding this niche cell type adds to our appreciation of stromal cells as active members of inflammatory pathology and supplements the roles LIF is already known to play in cancer. For instance, LIF is frequently overexpressed in many solid tumors including colorectal cancers, breast cancers, and skin cancers, and LIF overexpression in tumors correlates with poor prognosis of patients [59–61]. In mouse models, LIF blockade leads to reduced tumor progression and was able to synergize with immune checkpoint blockade to extend survival [62–65]. Our analysis of human tumor data hints that LIF may also have local effects supporting cancer-associated fibroblasts, a cell type that has recently drawn attention as key member of the tumor immune environment [66]. Collectively, we propose the parallel mechanisms of IL-1α and LIF that can synergize to activate splenic HSC niche to increase PMN production that may function in human cancers (Fig 7G).

Our data add depth and scope to the mechanisms by which cancers manipulate the host to generate a favorable immune environment for their growth, stretching as far up the differentiation hierarchy as primitive hematopoietic stem cells and their associated niche. One avenue that our paper focuses on is the cytokine axis established by tumor cells themselves. This focus uses an emerging classification of tumors by their functional effects that helps overcome heterogeneity both within and between tumor types and also makes comparisons of tumor pathology more congruous across species boundaries [67,68]. Studying tumor-derived cytokines also dovetails with the recent developments in understanding the reaction of HSPCs to inflammation [13,69–72]. While previous work has predominantly investigated cytokines and their impact on the BM microenvironment across different disease models, we were interested in how the spleen reacts to these inflammatory cytokines due to the organ’s increasing recognition as a site of reactive hematopoiesis. In fact, our data suggest that the spleen conducts a larger hematopoietic response to tumor-mediated systemic inflammation than the BM. Our data also add to the growing evidence supporting an active role in pathology played by HSPCs through inflammatory cytokine production [9,73]. Many cases of cytokine-induced changes to HSPCs result in myeloid lineage bias. This shift towards the production of myeloid cells benefits tumor growth while tending to harm cancer patients. Across multiple tumor types, including breast, colon, pancreatic, and gastric cancer, as well as a systematic review of all cancer types, a high neutrophil-to-lymphocyte ratio is an independent prognostic factor for survival [17–20]. In addition to increased quantity, myeloid cells produced in communication with cancer cells have unique qualities that help drive cancer pathology, such as MDSCs [9]. Our data expand the function of inflammatory cytokines produced by HSPCs beyond myeloid lineage biasing. Particularly, we provide data showing that TNFα expressed by HSPCs can regulate the function of their own niche. In concert with tumor-produced LIF that expands the quantity of splenic HSPC niche cells, tumor-derived IL-1α induces TNFα expression by HSPCs to alter niche function into favoring increased EMH. This paper uses IL-1R antagonism to hinder EMH response in our tumor model, hinting at the IL-1 family as a therapeutic target in cancer. Unlike IL-1 antagonism, the development of molecules to block LIF signal are ongoing and, based on our data, may prove to be a fruitful avenue of research. Together, these data warrant future studies addressing whether disruption of the local IL-1α/TNFα axis can impede EMH and how cell products of EMH induced by IL-1α and LIF impact the tumor microenvironment and cancer outcomes.

## Materials and methods

### Mice

Wild-type C57BL/6J mice (#000664), B6N.Cg-Tg(PDGFRa-cre/ERT)467Dbe/J (#018280), B6.SJL-Ptprca Pepcb/BoyJ (#002014), and B6.FVB-Tg(Cdh5-cre)7Mlia/J mice (#006137) were obtained from The Jackson Lab. MMTV-PyMT mice on a C57BL/6J background were a gift from Dr. M. Egeblad. *Lifr*-flox mice were obtained courtesy of Dr. Colin Steward [74]. All mice used in experimentation were female between the ages of 8 and 16 weeks and killed by CO_2_ asphyxiation followed by cervical dislocation unless otherwise stated. Animal husbandry, handling, and experimentation were approved by the Institutional Animal Care and Use Committee of Washington University School of Medicine under the protocol numbers 19–0961 and 22–0291.

### Mouse tumor models

MMTV-PyMT transgenic mice were used as a spontaneous model of breast cancer and were analyzed when evidence of peripheral neutrophilia was present, which was between 3 to 6 months. For tumor transplantation studies, 5 × 10^5^ PyMT-B6 tumor cells, 5 × 10^5^ LLC tumor cells, 2 × 10^6^ 1956, or 2.5 × 10^5^ PyMT-B6 gene knockout tumors cells were injected subcutaneously in a slurry of 1:1 EHS ECM growth factor-reduced gel (Corning, # 354230; Sigma, #E6909) to PBS into the flank of the mouse and harvested after 21 days for PyMT-B6, 16 or 21 days for LLC, 17 or 21 days for 1956, and 21 or 28 days for PyMT-B6 gene knockout experiments. PyMT-B6, wild-type and knockout, cells and LLC cells were grown in DMEM with penicillin/streptomycin, 10% fetal bovine serum, and 10 mM HEPES buffer. 1956 cells were grown in RPMI-1640 with penicillin/streptomycin, 10% fetal bovine serum, 100 mM sodium pyruvate, 7.5% v/v sodium bicarbonate, and 50 μM beta-mercaptoethanol. Supernatants were collected after 72 hours of incubation in culture starting after an overnight following passage, while lysates were harvested from culture dishes at the same time by a freeze–thaw cycle in cell culture media. Cell culture samples for ELISA were spun at 2,000*g* for 10 minutes, and the supernatant collected and used for ELISA measurement.

### Flow cytometry

Spleens were homogenized through a 100-μm filter. When analyzed for splenic ABS, spleens were digested for 30 minutes at 37C in 4 mL of 2.5 mg/mL Collagenase I and 2.5 mg/mL Dispase before passage over a 100-μm filter. BM from femur and tibias was ejected by centrifugation at 3,200*g* for 2 minutes at 4C. PB was collected by cheek bleed. RBCs were lysed when needed using ACK lysis buffer (Thermo Fisher, A10492-01). Cells were counted on an automated Nexcelom cell counter.

Cells were blocked with TruStain FcX PLUS anti-CD16/32 antibody (Biolegend, 156603) or anti-CD16/32 BV421 (Biolegend, clone 93) where appropriate before staining with antibodies followed by flow cytometry on a Gallios (Beckman Coulter) or a FACScan II (BD). When staining for intracellular cytokines, Cytofix/Cytoperm (BD, 554714) was used according to manufacturer’s instruction, and 1 μg/mL brefeldin A was maintained in the FACS buffer until fixation. Viability staining was added according to manufacturer’s instructions before beginning flow cytometry. Analysis was performed with FlowJo v10 software (Tree Star).

The following antibodies and reagents were purchased from BioLegend: anti-CD45.2 APC (clone 104), anti-CD11b APC-Cy7 (clone M1/70), anti-CD11b PE (clone M1/70), anti-Gr1 FITC (clone RB6-8C5), anti-Gr1 APC (clone RB6-8C5), anti-B220 PerCP/Cy5.5 (clone RA3-6B2), anti-B220 PerCP-Cy5.5 (clone RA3-6B2), anti-CD3e FITC (clone 145-2C11), anti-CD3e PE-Cy7 (clone 145-2C11), anti-Sca-1 APC (clone D7), anti-Sca-1 PerCP-Cy5.5 (clone D7), anti-CD45 AF700 (clone 30-F11), anti-CD45 BV421 (clone 30-F11), anti-c-Kit PE-Cy7 (clone 2B8), anti-c-Kit PE (clone 2B8), anti-VCAM-1 APC (clone 429), anti-PDGFRβ APC (clone APB5), anti-PDGFRα PE (clone APA5), 7-AAD dye (#420404), anti-IL-7R PE-Cy7 (clone A7R34), anti-CD84 PE (clone mCD84.7), anti-Ly6C BV510 (clone HK1.4), anti-Ly6G FITC (clone 1A8), anti-CD11b APC/Cy7 (clone M1/70), streptavidin PerCP-Cy5.5 (#405214), streptavidin BV421 (#405225), streptavidin APC (#405207), and biotin anti-lineage (#133307). Anti-CD34 FITC (clone RAM34) was purchased from Thermo. Anti-CD45.1 PE (clone A20) was purchased from BD Biosciences. Anti-KITL biotin (#102501) and biotinylated goat IgG control (#105601) were purchased from R&D Systems.

Cell type delineations were made as follows: KSL cells were gated as CD45^+^/Lineage^−^/c-Kit^+^/Sca-1^+^; GMP cells were gated as CD45^+^/Lineage^−^/c-Kit^+^/Sca-1^−^/CD16/32^+^/CD34^+^; CLP cells were gated as CD45^+^/Lineage^−^/c-Kit^−^/Sca-1^+^/IL-7R^+^; PMN-MDSC cells were gated as CD45^+^/CD84^+^/CD11b^+^/Ly6G^+^; M-MDSC cells were gated as CD45^+^/CD84^+^/ CD11b^+^/Ly6G^−^/Ly6C^hi^; ABS cells were gated as CD45^−^/PDGFRα^+^/PDGFRβ^+^.

### Colony-forming assay

PB or the full contents of ABS:hematopoietic progenitor coculture 24-wells were plated into complete methylcellulose media (Stem Cell Technologies, M3434). Colonies were scored 7 to 14 days after plating.

### Bone marrow transplant

For splenocyte transplantation, CD45.2 mice were irradiated with 9.5 Gy and 1 × 10^6^ splenocytes from CD45.1 control or CD45.1 tumor-bearing animals were injected intravenously by the retroorbital route 24 hours after irradiation. For niche function studies, CD45.2 mice were irradiated with 9.5 Gy and CD45.1 hematopoietic cells were isolated from cell culture with or without ABS cells and injected intravenously by the retroorbital route 24 hours after irradiation. Mice were monitored daily for mortality or signs of severe morbidity up to 28 days. Mice were maintained until mortality to evaluate the long-term reconstitution potential.

### Splenectomy

Splenectomies and sham surgeries were conducted courtesy of the Hope Center Animal Surgery Core, Washington University School of Medicine. After a week recovery period, mice were injected with PyMT-B6 tumor cells as detailed above.

### Single-cell RNA-sequencing and analysis

Spleens were minced and digested in 1 mg/mL Collagenase Type IV + 0.25mg/mL DNase I. BM was removed by centrifugation as detailed above and digested. Digestion was quenched then filtered through a 100-μm filter. Cells were pelleted, counted, and aliquoted. TruStain FcX PLUS was used to block samples, then biotin anti-lineage antibodies were used to stain lineage cells. After washing, strepavidin magnetic beads (NEB, S1420S) were used to deplete lineage positive cells. Remaining cells were pelleted and then stained with streptavidin BV605 (Biolegend, #405229), anti-CD45 AF700, anti-PDGFRα APC, anti-CD51 PE (Biolegend, clone RMV-7), anti-CD31 PE-Cy7 (Biolegend, clone 390), anti-Sca-1 PerCP-Cy5.5 (Biolegend, clone D7), and anti-c-Kit FITC (Biolegend, clone 2B8). Cells were then washed into holding buffer (0.04% BSA in PBS), stained with DAPI, and sorted on a high modified MoFlo into 5 populations: Live/Lin^−^/CD45^+^/c-Kit^+^/Sca-1^+^, Live/Lin^−^/CD45^+^/c-Kit^+^/Sca-1^−^, Live/Lin^−^/CD45^−^/CD31^+^, Live/Lin^−^/CD45^−^/CD31^−^, Live/Lin^−^/CD45^−^/CD31^−^/CD51^+^, Live/Lin^−^/CD45^−^/CD31^−^/CD51^−^. These populations were combined at equal ratios and submitted for 10X Genomics 3′ v3.1 Chemistry sample preparation and sequencing on a NovaSeq6000 at the Genome Technology Access Center.

Cell Ranger (10X Genomics, Pleasanton, CA) with default settings demultiplexed, aligned, filtered, and counted barcodes and UMIs. SoupX preprocessing was used to remove ambient RNA contamination at a contamination fraction of 10% [75]. Filtered outputs were imported into R v4.0.5 using Seurat v3.2.3, and barcodes with fewer than 350 unique genes were excluded. Seurat objects from the 4 experiment groups were merged, and an SCT transformation with a variable feature count of 20,000 was performed on the resulting object. [76,77] The dimensions of the object were reduced using RunPCA with principal coordinates equal to 50. UMAP coordinates were calculated using all 50 PCA dimensions and a minimum distance of 0.05. FindNeighbors function was used to compute nearest neighbors using all 50 PCA dimensions, and FindClusters function at a resolution of 1.2 was used to compute cell clusters. Markers for each cluster were calculated using FindAllMarkers function with a minimum percentage of 0.1.

For reanalysis of a publically available scRNA-seq dataset of BM niche cells [49], data were downloaded from GSE108891 on Gene Expression Omnibus. Raw counts files for GSM2915575, GSM2915576, GSM2915577, and GSM3330917 were imported into R using Seurat 3.2.3, and barcodes with fewer than 500 unique genes were excluded. Seurat objects from the 4 experiment groups were merged, and an SCT transformation with a variable feature count of 8,000 was performed on the resulting object [76,77]. The dimensions of the object were reduced using RunPCA with principal coordinates equal to 20. UMAP coordinates were calculated using all 20 PCA dimensions and a minimum distance of 0.05. FindNeighbors function was used to compute nearest neighbors using all 20 PCA dimensions, and FindClusters function at a resolution of 0.2 was used to compute cell clusters. Markers for each cluster were calculated using FindAllMarkers function on default settings.

### Magnetic bead isolation and quantitative reverse transcriptase analysis

TruStain FcX PLUS antibody was used to block samples, then biotin anti-lineage antibodes and biotin anti-Flk1 (Biolegend, clone 89B3A5) antibody were used to stain cells. After washing, strepavidin magnetic beads were used to bind the stained cells. Positive cells were depleted by 2 rounds of magnetic selection. Depleted cells were pelleted and stained with anti-CD34 FITC and anti-FITC biotin (Biolegend, clone FIT-22). Cells were washed, pelleted, and resuspended before adding streptavidin magnetic beads. After incubation, the tubes were placed on the magnet and the supernatant removed. Using an RNeasy Kit Micro (Qiagen, #74004), RLT buffer was used to lyse the cells before proceeding with RNA isolation according to manufacturer’s instructions. qScript cDNA SuperMix (QuantaBio, 95048–100) was used to produce cDNA before running RT-qPCR with 2x SYBR Green qPCR Master Mix (BiMake, B21203) according to manufacturer instructions. Primers sequences were as follows: *Tnf* forward—CCCTCACACTCAGATCATCTTCT, reverse—GCTACGACGTGGGCTACAG; *Cxcl2* forward–CCAACCACCAGGCTACAGG, reverse–GCGTCACACTCAAGCTCTG; *Nfkbia* forward–TGAAGGACGAGGAGTACGAGC, reverse–TTCGTGGATGATTGCCAAGTG; *Nfkbiz* forward–GCTCCGACTCCTCCGATTTC, reverse–GAGTTCTTCACGCGAACACC; *Mki67* forward–ATCATTGACCGCTCCTTTAGGT, reverse–GCTCGCCTTGATGGTTCCT; *Il1r1* forward–GTGCTACTGGGGCTCATTTGT, reverse–GGAGTAAGAGGACACTTGCGAAT; *Hprt* forward–TCAGTCAACGGGGGACATAAA, reverse–GGGGCTGTACTGCTTAACCAG.

### ELISA and multiplex protein assay

ELISA kits for IL-1α (Abcam, ab199076), CXCL1 (R&D, DY453-05), and CXCL12 (Abcam, ab100741) were used according to manufacturers’ instructions. LIF serum samples were analyzed using the Abcam, ab238261, while all other sample types were analyzed using R&D, DY449. Serum samples from MMTV-PyMT mice and littermate controls and from PyMT-B6 tumor-bearing mice and PBS-injected controls for quantification of TNFα were sent to Eve Technologies (Calgary, AB, Canada) and assayed using the 32-plex or 44-plex Mouse Discovery assay. Results from Eve Technologies were imported into R, log10 normalized, and plotted using the heatmap.2 function in the gplots package.

### In vivo cytokine or antibody injection

TNFα (Peprotech, 315-01A) and IL-1α (Peprotech, 211-11A) were purchased, resuspended according to manufacturer’s instructions. For TNFα and IL-1α experiment, 2 μg and 0.5 μg or 0.2 μg per mouse were injected retroorbitally, respectively. Mice were analyzed 24 hours later.

For antibody blockade of IL-1R, mice given PyMT-B6 tumors as described above were injected IP with 200 μg IL-1R antibody (InvivoMab, #BE0256) or isotype control (InvivoMab, #BE0091), resuspended according to manufacturer’s instruction every third day beginning on day 3 and finishing on day 18 with the animals killed on day 21 for analysis.

### CRISPR-Cas9 gene deletion in PyMT-B6 Cells

PyMT-B6 cells were seeded and then grown overnight to around 70% confluence before adding TrueCut Cas9 Protein v2 (Thermo, A36497), Lipofectamine CRISPRMAX Cas9 Transfection Reagent, and TrueGuide Synthetic sgRNA (Thermo, #A35533) according to manufacturer’s instructions. Guide RNAs from the manufacturer’s catalog were selected to be positioned in the earliest exon shared by all known isoforms and to minimize the distance between the 2 cut sites. Both guides were incubated with the cells during lipofection. After lipofection, cells with single cell cloned. Each clone was tested for deletion of the gene by ELISA, Sanger sequencing, and gel electrophoresis when applicable. *Csf3* was deleted initially, then a successful clone was used as the parental line for subsequent deletion of *Lif* or *Il1a*. These knockout cell lines were injected in vivo as described above.

### Splenic stromal cell isolation, culture, and coculture with hematopoietic progenitors

Spleens were minced and plated on gelatin-coated plates. Growth media for cells was alpha-MEM with 10% FBS, 1x Glutamax, 10 mM HEPES buffer, 100 μg/mL Primocin (InVivogen, ant-pm), and 5 ng/mL heat-stable FGF2 (Gibco, PHG0368). After 72 hours, nonadherent tissue was gently removed. Media was changed every 2 to 3 days thereafter until the culture was 100% confluent. Cells were passaged using CellStripper and plated without gelatin coating. For flow cytometry experiments involving membrane KITL staining, cells were lifted using CellStripper and stained. For other flow cytometry experiments and cytokine stimulation, cells were lifted with Trypsin–EDTA. For LIF stimulation experiments, cells were plated at 5,000 cells/cm^2^, grown overnight in growth media, then changed to growth media without heat-stable FGF2 with or without 20 ng/mL LIF (Peprotech, 250–02). Media was changed after 2 days, and the RNA was harvested on the third day. For TNFα stimulation experiments, cells were plated at 10,000 cell per cm^2^, grown over night in growth media, then changed to growth media with or without 2.5 ng/mL of TNFα (Peprotech, 315-01A). Cells or supernatant were harvested after 24 hours for flow cytometry or ELISA, respectively.

For coculture with hematopoietic stem and precursor cells, splenic stromal cells were plated and grown until confluence before 5,000 live c-Kit^+^ Lineage^−^ cells were sorted and transferred into individual 24-wells with or without a stromal monolayer. Cocultures were then grown for 7 days before passage or usage in an experiment as specified. The same media was used for coculturing as was used for monoculture of splenic stromal cells.

### Lentiviral particle production and administration

Murine LIF ORF (NM_008501.2) was purchased from GenScript and cloned into the pCSII-EF1α-IRES2-bsr lentiviral backbone. Lentiviral packaging plasmid psPAX2 (Addgene, plasmid #12260) and VSV-G envelope expressing plasmid PMD2.G (Addgene, plasmid #12259) were gifts from Didier Trono. 293FT cells were transfected with lentiviral DNA using the calcium phosphate method. Virus was concentrated from media using PEG Virus Precipitation Kit (Sigma). Viral titer was determined by QuickTiter Lentivirus Associated HIV p24 Titer Kit (Cell Biolabs, INC). Mice were infected by tail vein injection with 4 × 10^9^ viral particles before killing on day 7 for immunofluorescence experiments or on day 10 for all other experiments.

### Immunofluorescence, bright-field, and confocal microscopy

For immunofluorescence, spleens were removed from animals and directly embedded by freezing into NEG-50 media. Using 4% PFA in PBS, 6-μm sections were fixed then permeabilized in 0.5% Triton-X100 in PBS before blocking with 1% BSA/ 5% donkey serum in PBS. Sections were stained with primary antibodies overnight and then stained with secondary antibodies for 1 hour. Primary antibodies, anti-PDGFRa (AB Online, # ABIN726620) and anti-Ki67 (Biolegend, clone 16A8), were diluted 1:200 for staining. Secondary antibodies were donkey anti-rabbit AF488+ (Thermo Fisher, # A32790) and donkey anti-rat AF594+ (Thermo Fisher, #A21209). Sections were quenched using ReadyProbes Tissue Autofluorescence Quenching Kit (Thermo Fisher, R37630) according to manufacturers’ instructions before staining with DAPI and mounting with ProLong Diamond Antifade Mountant (Thermo Fisher, P36970). Slides were sealed and imaged using a Zeiss AxioImager Z2 at the Washington University Center for Cellular Imaging using Zen Blue v.3 for image acquisition and processing. Images were counted manually.

For bright-field microscopy, day 7 stromal:hematopoietic cocultures were imaged live on an ACCU-SCOPE EXI-600 inverted microscope. Images were processed using ImageJ [78].

For confocal microscopy, spleens were removed from animals and fixed in 4% PFA (Electron Microscope Sciences, #15710-S) with PBS for 72 hours. Spleens were washed overnight in PBS and then sectioned by Vibratome to 300 μm. Sections were then cleared using 10% w/v CHAPS and 25% v/v N-Methyldiethanoamine in PBS for 48 hours before washing with PBS followed by 72 hours of blocking 5% donkey serum (Sigma, #D9663) in PBS. Primary antibodies, anti-PDGFRa (AB Online, # ABIN726620), anti-Kitl (R&D, #AB-455-NA), and anti-c-Kit (Biolegend, clone 2B8) were then stained at a 1:200 dilution for 72 hours. Sections were washed with PBS overnight before staining at 1:250 with secondary antibodies, donkey anti-rat AF647+ (Thermo, # A48272), donkey anti-rabbit AF555 (Thermo, A-31572), and donkey anti-Goat AF405+ (Thermo, # A48259). After secondary staining, sections were washed overnight with PBS before dehydration with increasing concentrations of ethanol—50%, 70%, 95%, and 95%—for at least 2 hours each before incubation with a methyl salicylate solution (Sigma-Aldrich, M6752) for 30 to 60 minutes in a custom metal chamber with 0.2 mm coverslip glass bottom. Tissue sections were then imaged at 1.5 μm optical sections using a 7-laser inverted Leica SP8 microscope with full spectral hybrid detectors. All image collection was performed using Leica LAS X software, and analysis was performed using Leica LAS X or Imaris (Bitplane) v8 and v9 software. Images shown are maximum intensity projections of 8 sections representing 10.5 μm in depth.

### Human tissue datasets and xCell analysis

Transcriptomic data of tumor and normal samples were downloaded from The Cancer Genome Atlas (TCGA), Therapeutically Applicable Research To Generate Effective Treatments (TARGET), and Genotype-Tissue Expression (GTEx) consortiums were downloaded using the UCSC Xena portal (https://xena.ucsc.edu/). Normalized RSEM expected counts were logged for visualization and statistical purposes.

A signature-based deconvolution pipeline, xCell [79], was used to identify enrichment of stromal populations in the tumor microenvironment. Gene length normalized TPM data from TCGA was downloaded from the UCSC Xena portal and was used as an input into xCell for stromal cell deconvolution. Patients were grouped into quartiles by LIF expression and compared across subgroups.

### Quantification and statistical analysis

Statistical analyses were performed using GraphPad Prism 9 software (GraphPad Software, San Diego, CA). *P* values were calculated using unpaired *t* tests (two-tailed) unless otherwise indicated in the figure legends. *P* values less than 0.05 was considered statistically significant and displayed above the comparison bars in figures. Each figure represents at least 2 independent experiments and are presented together unless otherwise specified. Error bars show the standard error of the mean for each sample.

## Supporting information

S1 Fig Genetic PyMT, 1956, and LLC tumor models induce splenic hematopoiesis. (**A**) PMNs in the PB as percent of CD45^+^ cells in female mice between the ages of 3 to 6 months with spontaneous mammary tumors in the MMTV-PyMT tumor model compared with nontumor bearing, littermate controls (*n =* 9). (**B**–**I**) In mice with MMTV-PyMT mammary tumors compared with littermate controls, splenic weight (*n* = 11–12), splenic cellularity (**C**, *n* = 11–12), KSL cells as a fraction of total splenic CD45^+^ cells (**D**, *n* = 4–5), GMP cells as a fraction of total splenic CD45^+^ (**E**, *n* = 4–5), CLP cells as a fraction of total splenic CD45^+^ cells (**F**, *n* = 4–5), BM cellularity per leg (**G**, *n* = 11–12), KSL cells as a fraction of total BM CD45^+^ cells (**H**, *n* = 4–5), GMP cells as a fraction of total BM CD45+ cells (**I**, *n* = 4–5). (**J**–**M**) Twenty-one days after injection of 5 × 10^5^ PyMT-B6 tumor cells injected subcutaneously compared to control animals injected with PBS, PMN-MDSC cells as a fraction of PB CD45^+^ cells (**J**, *n* = 4, 1 independent experiment), PMN-MDSC cells as a fraction of splenic CD45^+^ cells (**K**, *n* = 4, 1 independent experiment), M-MDSC cells as a fraction of PB CD45^+^ cells (**L**, *n* = 4, 1 independent experiment), M-MDSC cells as a fraction of splenic CD45^+^ cells (**M**, *n* = 4, 1 independent experiment). (**N**) Percent of donor-derived PB CD45^+^ 1 month after transplantation of splenocytes from mice with 21 days of PyMT-B6 tumor into 9.5 Gy irradiated mice (*n =* 15). (**O**, **P**) Seventeen days after injection of 2 × 10^6^ 1956 tumor cells injected subcutaneously compared to control animals injected with PBS, PMNs in the PB as a percent of total leukocytes (**O**, *n* = 8), GMP cells per spleen (**P**, *n* = 8). (Q, R) Sixteen days after injection of 5 × 10^5^ LLC tumor cells injected subcutaneously compared to control animals injected with PBS, PMNs in the PB as a percent of total leukocytes (**Q**, *n* = 6–7), GMP cells per spleen (**R**, *n* = 6–7). Processed data for this figure can be found in S1 Data. BM, bone marrow; CLP, common lymphoid progenitor; GMP, granulocyte–monocyte precursor; KSL, Kit^+^/Sca-1^+^/Lineage^−^; LLC, Lewis lung carcinoma; MDSC, myeloid-derived suppressor cell; MMTV, murine mammary tumor virus; PB, peripheral blood; PBS, phosphate buffered saline; PMN, polymorphonuclear neutrophil; PyMT, polyomavirus middle T antigen.
(TIF)

S2 Fig c-Kit^+^Sca-1^+^CD34^+^ HSPCs express IL-1R. (**A**–**I**) From analysis of myeloid progenitor clusters within our scRNA-seq data of BM and spleen cells with or without PyMT-B6 tumor, table with cluster assignments and marker genes (**A**), UMAP projection colored by cluster identity (**B**), by expression of *Kit* (**C**), *Ly6a* (**D**), *Cd34* (**E**), *Cd48* (**F**), *Slamf1* (**G**), *Tnf* (**H**), *Il1r1* (**I**). (**J**) Twenty-one days after injection of 2 × 10^6^ 1956 tumor cells, 5 × 10^5^ LCC tumor cells, or 5 × 10^5^ PyMT-B6 tumor cells, injected subcutaneously compared to control animals injected with PBS, percent of KSL with positive TNFα staining (*n* = 4–5, 1 independent experiment, significance assigned by one-way ANOVA). Processed data for this figure can be found in S1 Data. BM, bone marrow; HSPC, hematopoietic stem and progenitor cell; KSL, Kit^+^/Sca-1^+^/Lineage^−^; LLC, Lewis lung carcinoma; PBS, phosphate buffered saline; PyMT, polyomavirus middle T antigen; scRNA-seq, single-cell RNA-sequencing.
(TIF)

S3 Fig Diverse tumor models induce inflammatory splenic HSPC phenotypes. (**A**, **B**) In mice 24 hours after IV injection of 500 ng IL-1α or vehicle, KSL cells as a fraction of total splenic CD45^+^ cells (**A**, *n =* 7), GMP cells as a fraction of total splenic CD45^+^ cells (**B**, *n* = 7). (**C**, **D**) Twenty-eight days after subcutaneous injection of 2.5 × 10^5^ PyMT-B6 ΔG-CSF parental cells or ΔG-CSFΔ IL-1α cells, PMNs in the PB as a percent of total leukocytes (**C**, *n =* 7–13), KSL cells per spleen (**D**, *n* = 7–13). (**E**–**G**) Twenty-one days after injection of 2.5 × 10^5^ PyMT-B6 parental tumor cells or ΔG-CSF tumor cells injected subcutaneously compared to control animals injected with PBS, PMNs in the PB as a percent of total leukocytes (**E**, *n* = 4, 1 independent experiment), KSL cells as a fraction of total splenic CD45^+^ cells (**F**, *n* = 4, 1 independent experiment), GMP cells as a fraction of total splenic CD45^+^ cells (**G**, *n* = 4, 1 independent experiment). Processed data for this figure can be found in S1 Data. GMP, granulocyte–monocyte precursor; HSPC, hematopoietic stem and progenitor cell; IV, intravenous; KSL, Kit^+^/Sca-1^+^/Lineage^−^; PB, peripheral blood; PBS, phosphate buffered saline; PMN, polymorphonuclear neutrophil; PyMT, polyomavirus middle T antigen.
(TIF)

S4 Fig BM KITL is expressed by PDGFRα^+^/β^+^ stromal cells and Cdh5^+^Sca-1^+^ endothelial cells. (**A**–**I**) From reanalyzed scRNA-seq data of Tikhonova and colleagues of BM niche cell types [49], table with cluster assignments and marker genes (**A**), violin plot of expression of *Cxcl12* (**B**), *Lepr* (**C**), *Cdh5* (**D**), *Ptprc* (**E**), *Kit* (**F**), *Ly6a* (**G**), *Bglap* (**H**), and *Tnfrsf1b* (**I**). (**J**–**L**) Hematopoietic and stromal cocultures after 7 days, representative bright-field image of coculture at 4× magnification (**J**), representative bright-field image of coculture at 20× magnification (**K**), representative flow cytometric plot of Live/CD45^+^ cells with KSL cells as a percentage of Lineage^−^ cells (**L**). (**M**) Percent of donor-derived PB CD45^+^ 1 month after transplantation of hematopoietic and stromal coculture cells into 9.5 Gy irradiated mice (*n* = 17). Processed data for this figure can be found in S1 Data. The raw flow cytometry data, gating schema, and staining profile relevant to S4L Fig are deposited on Flow Repository under accession number FR-FCM-Z628. BM, bone marrow; KSL, Kit^+^/Sca-1^+^/Lineage^−^; PB, peripheral blood; scRNA-seq, single-cell RNA-sequencing.
(TIF)

S5 Fig Tumor LIF impacts predominantly impacts the splenic niche. (**A**) Heatmap showing z-scores of log-normalized expression of 44 cytokines from the serum of MMTV-PyMT^+^, tumor-bearing animals (PyMT), or age-matched, nontumor-bearing littermates (WT) (*n =* 2). (**B**–**E**) In mice with 10 days of LIF overexpression or empty vector control, KSL cells as a fraction of total BM CD45+ cells (**B**, *n* = 7–8), GMP cells as a fraction of total BM CD45+ cells (**C**, *n* = 7–8), KSL cells per leg (**D**, *n* = 7–8), GMP cells per leg (**E**, *n* = 7–8). (**F**, **G**) Twenty-eight days after subcutaneous injection of 2.5 × 10^5^ PyMT-B6 ΔG-CSF parental cells or ΔG-CSFΔLIF cells, PMNs in the PB as a percent of total leukocytes (**F**, *n* = 12), GMP cells per spleen (**G**, *n* = 12). Processed data for this figure can be found in S1 Data. The raw cytokine profiling results used to generate S5A Fig are available as S2 Data file. BM, bone marrow; GMP, granulocyte–monocyte precursor; KSL, Kit^+^/Sca-1^+^/Lineage^−^; LIF, leukemia inhibitory factor; MMTV, murine mammary tumor virus; PB, peripheral blood; PMN, polymorphonuclear neutrophil; PyMT, polyomavirus middle T antigen; WT, wild type.
(TIF)

S6 Fig Tumor LIF increases local stromal cells but not endothelial cells. (**A**, **B**) In day 12 postpartum LIFR^flox^ mice with PDGFRα-Cre^+^ or PDGFRα-Cre^−^ littermates, body weight (**A**, *n* = 6–9, contains male mice), splenic weight as a fraction of total body weight (**B**, *n* = 6–9, contains male mice). (**C**) Representative immunofluorescence image of the spleen after LIF overexpression with PDGFRα^+^ cells in green, Ki67^+^ nuclei in red, and DAPI^+^ nuclei in blue. (**D**) Representative confocal image of spleen after LIF overexpression with PDGFRα^+^ cells in green and c-Kit^+^ cells in blue. (**E**, **F**) Enrichment of fibroblasts (**E**) and endothelial cells (**F**) as calculated by xCell from RNA-seq data of human tumors split by top and bottom quartile of LIF expression (*n* = 416–417). Processed data for this figure can be found in S1 Data. LIF, leukemia inhibitory factor.
(TIF)

S7 Fig IL-1Α and LIF coexpression is common in human tumor types. (**A**–**C**) RNA-seq expression of *IL1A*, *LIF*, and *CSF3* expression in tumor compared to normal tissue for bile duct (**A**, *n* = 9–36), brain (**B**, *n* = 689–1,146), and gastric (**C**, *n* = 210–414) tumors. Processed data for this figure can be found in S1 Data. LIF, leukemia inhibitory factor; RNA-seq, RNA-sequencing.
(TIF)

S1 Data Excel spreadsheet containing all the processed data used to generate the graphs presented in this manuscript. This spreadsheet contains the processed data used to generate the graphs and statistics in the manuscript and is divided by originating figures into different sheets.
(XLSX)

S2 Data Excel spreadsheet containing the raw cytokine profiling data received from Eve Technologies that was processed into S5A Fig. This spreadsheet contains the raw cytokine profiling data from Eve Technologies used to generate the heatmap in the S5A Fig of the manuscript.
(XLSX)

## Abbreviations

BM: bone marrow
CBM: control BM
CLP: common lymphoid progenitor
EMH: extramedullary hematopoiesis
GMP: granulocyte–monocyte precursor
GTEx: Genotype-Tissue Expression
HSPC: hematopoietic stem and progenitor cell
KSL: Kit^+^/Sca-1^+^/Lineage^−^
LIF: leukemia inhibitory factor
LIFR: LIF receptor
LLC: Lewis lung carcinoma
MDSC: myeloid-derived suppressor cell
MMTV: murine mammary tumor virus
PB: peripheral blood
PBS: phosphate buffered saline
PMN: polymorphonuclear neutrophil
PyMT: polyomavirus middle T antigen
RT-qPCR: reverse transcription quantitative PCR
scRNA-seq: single-cell RNA-sequencing
TARGET: Therapeutically Applicable Research To Generate Effective Treatments
TBM: tumor-bearing BM
TCGA: The Cancer Genome Atlas
TS: tumor-bearing spleen
