# Supplementary figures and images for: Tumor-derived interleukin-1α and leukemia inhibitory factor promote extramedullary hematopoiesis
Source: PLoS Biol. 2023 May 3;21(5):e3001746. doi: 10.1371/journal.pbio.3001746 (PMC10155962; doi:10.1371/journal.pbio.3001746)

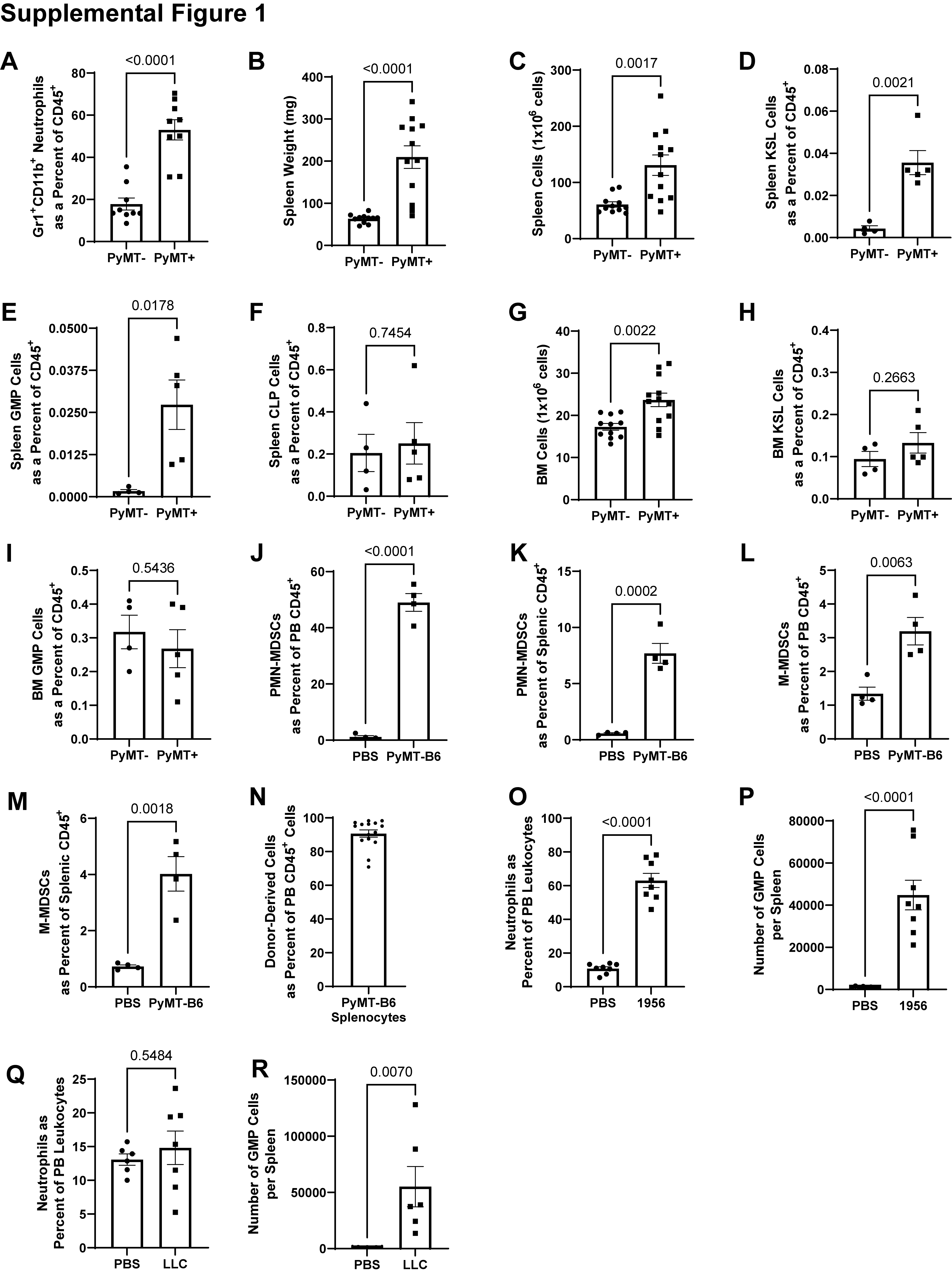

Supplement: S1 Fig — (A) PMNs in the PB as percent of CD45+ cells in female mice between the ages of 3 to 6 months with spontaneous mammary tumors in the MMTV-PyMT tumor model compared with nontumor bearing, littermate controls (n = 9). (B–I) In mice with MMTV-PyMT mammary tumors compared with littermate controls, splenic weight (n = 11–12), splenic cellularity (C, n = 11–12), KSL cells as a fraction of total splenic CD45+ cells (D, n = 4–5), GMP cells as a fraction of total splenic CD45+ (E, n = 4–5), CLP cells as a fraction of total splenic CD45+ cells (F, n = 4–5), BM cellularity per leg (G, n = 11–12), KSL cells as a fraction of total BM CD45+ cells (H, n = 4–5), GMP cells as a fraction of total BM CD45+ cells (I, n = 4–5). (J–M) Twenty-one days after injection of 5 × 10^5^ PyMT-B6 tumor cells injected subcutaneously compared to control animals injected with PBS, PMN-MDSC cells as a fraction of PB CD45+ cells (J, n = 4, 1 independent experiment), PMN-MDSC cells as a fraction of splenic CD45+ cells (K, n = 4, 1 independent experiment), M-MDSC cells as a fraction of PB CD45+ cells (L, n = 4, 1 independent experiment), M-MDSC cells as a fraction of splenic CD45+ cells (M, n = 4, 1 independent experiment). (N) Percent of donor-derived PB CD45+ 1 month after transplantation of splenocytes from mice with 21 days of PyMT-B6 tumor into 9.5 Gy irradiated mice (n = 15). (O, P) Seventeen days after injection of 2 × 10^6^ 1956 tumor cells injected subcutaneously compared to control animals injected with PBS, PMNs in the PB as a percent of total leukocytes (O, n = 8), GMP cells per spleen (P, n = 8). (Q, R) Sixteen days after injection of 5 × 10^5^ LLC tumor cells injected subcutaneously compared to control animals injected with PBS, PMNs in the PB as a percent of total leukocytes (Q, n = 6–7), GMP cells per spleen (R, n = 6–7). Processed data for this figure can be found in S1 Data. BM, bone marrow; CLP, common lymphoid progenitor; GMP, granulocyte–monocyte precursor; KSL, Kit+/Sca-1+/Lineage−; LLC, [file pbio.3001746.s001.tif]

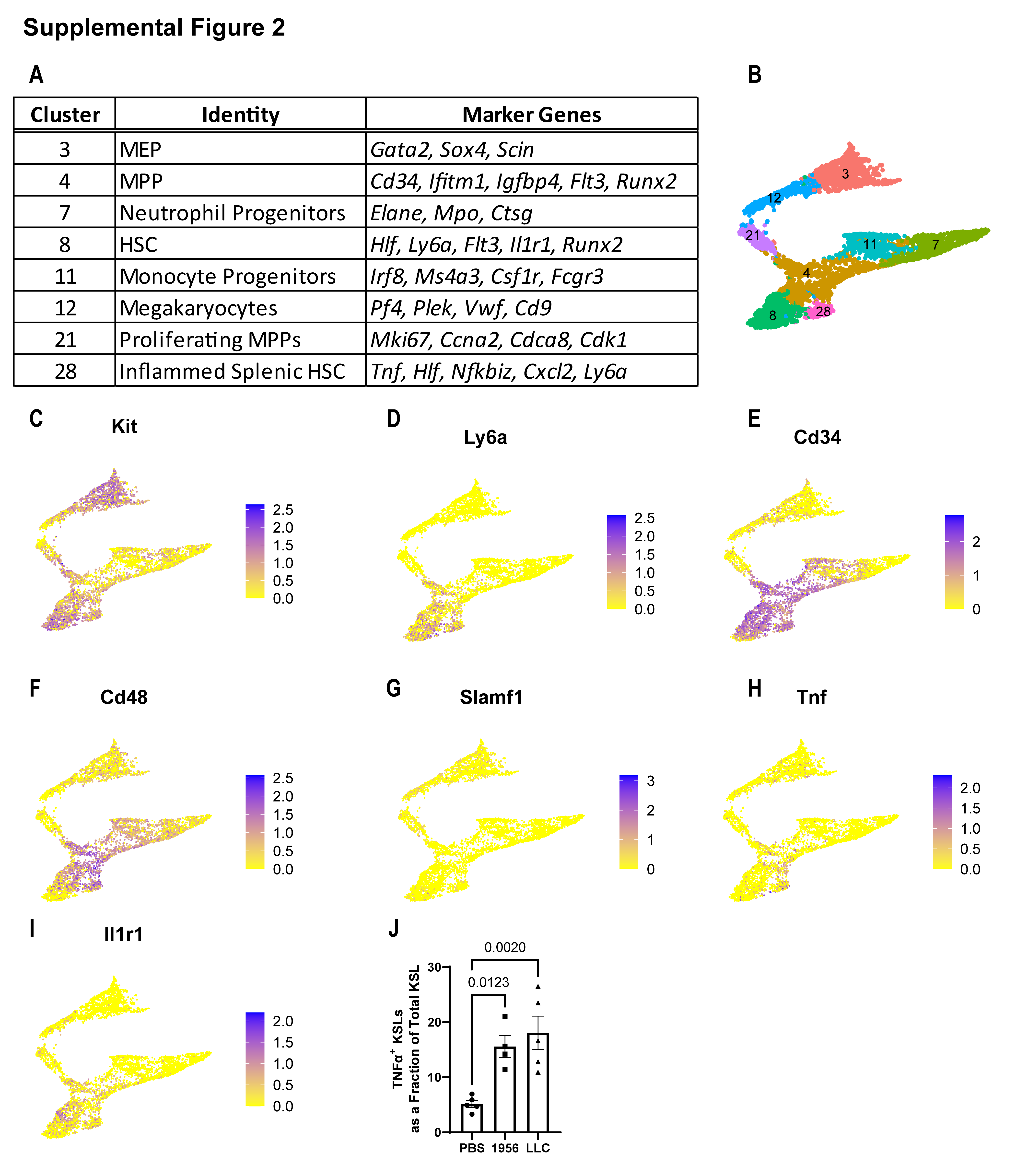

Supplement: S2 Fig — (A–I) From analysis of myeloid progenitor clusters within our scRNA-seq data of BM and spleen cells with or without PyMT-B6 tumor, table with cluster assignments and marker genes (A), UMAP projection colored by cluster identity (B), by expression of Kit (C), Ly6a (D), Cd34 (E), Cd48 (F), Slamf1 (G), Tnf (H), Il1r1 (I). (J) Twenty-one days after injection of 2 × 10^6^ 1956 tumor cells, 5 × 10^5^ LCC tumor cells, or 5 × 10^5^ PyMT-B6 tumor cells, injected subcutaneously compared to control animals injected with PBS, percent of KSL with positive TNFα staining (n = 4–5, 1 independent experiment, significance assigned by one-way ANOVA). Processed data for this figure can be found in S1 Data. BM, bone marrow; HSPC, hematopoietic stem and progenitor cell; KSL, Kit+/Sca-1+/Lineage−; LLC, Lewis lung carcinoma; PBS, phosphate buffered saline; PyMT, polyomavirus middle T antigen; scRNA-seq, single-cell RNA-sequencing. (TIF) [file pbio.3001746.s002.tif]

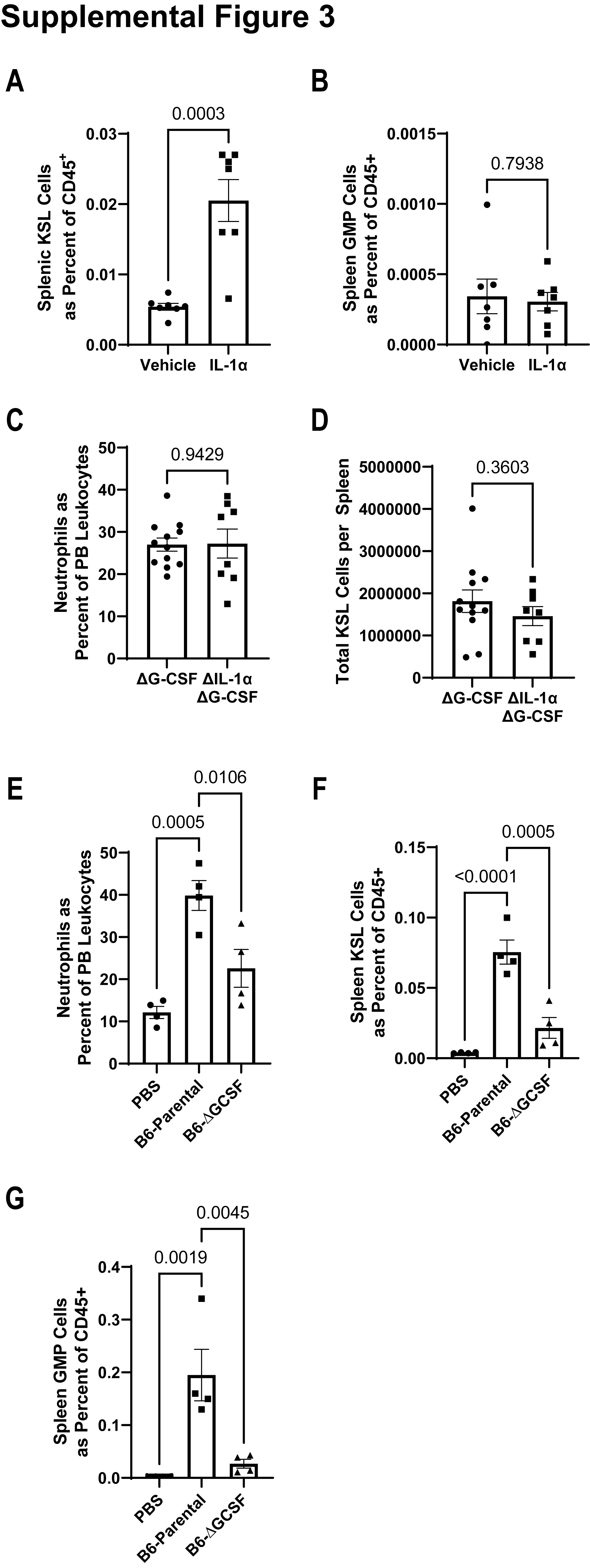

Supplement: S3 Fig — (A, B) In mice 24 hours after IV injection of 500 ng IL-1α or vehicle, KSL cells as a fraction of total splenic CD45+ cells (A, n = 7), GMP cells as a fraction of total splenic CD45+ cells (B, n = 7). (C, D) Twenty-eight days after subcutaneous injection of 2.5 × 10^5^ PyMT-B6 ΔG-CSF parental cells or ΔG-CSFΔ IL-1α cells, PMNs in the PB as a percent of total leukocytes (C, n = 7–13), KSL cells per spleen (D, n = 7–13). (E–G) Twenty-one days after injection of 2.5 × 10^5^ PyMT-B6 parental tumor cells or ΔG-CSF tumor cells injected subcutaneously compared to control animals injected with PBS, PMNs in the PB as a percent of total leukocytes (E, n = 4, 1 independent experiment), KSL cells as a fraction of total splenic CD45+ cells (F, n = 4, 1 independent experiment), GMP cells as a fraction of total splenic CD45+ cells (G, n = 4, 1 independent experiment). Processed data for this figure can be found in S1 Data. GMP, granulocyte–monocyte precursor; HSPC, hematopoietic stem and progenitor cell; IV, intravenous; KSL, Kit+/Sca-1+/Lineage−; PB, peripheral blood; PBS, phosphate buffered saline; PMN, polymorphonuclear neutrophil; PyMT, polyomavirus middle T antigen. (TIF) [file pbio.3001746.s003.tif]

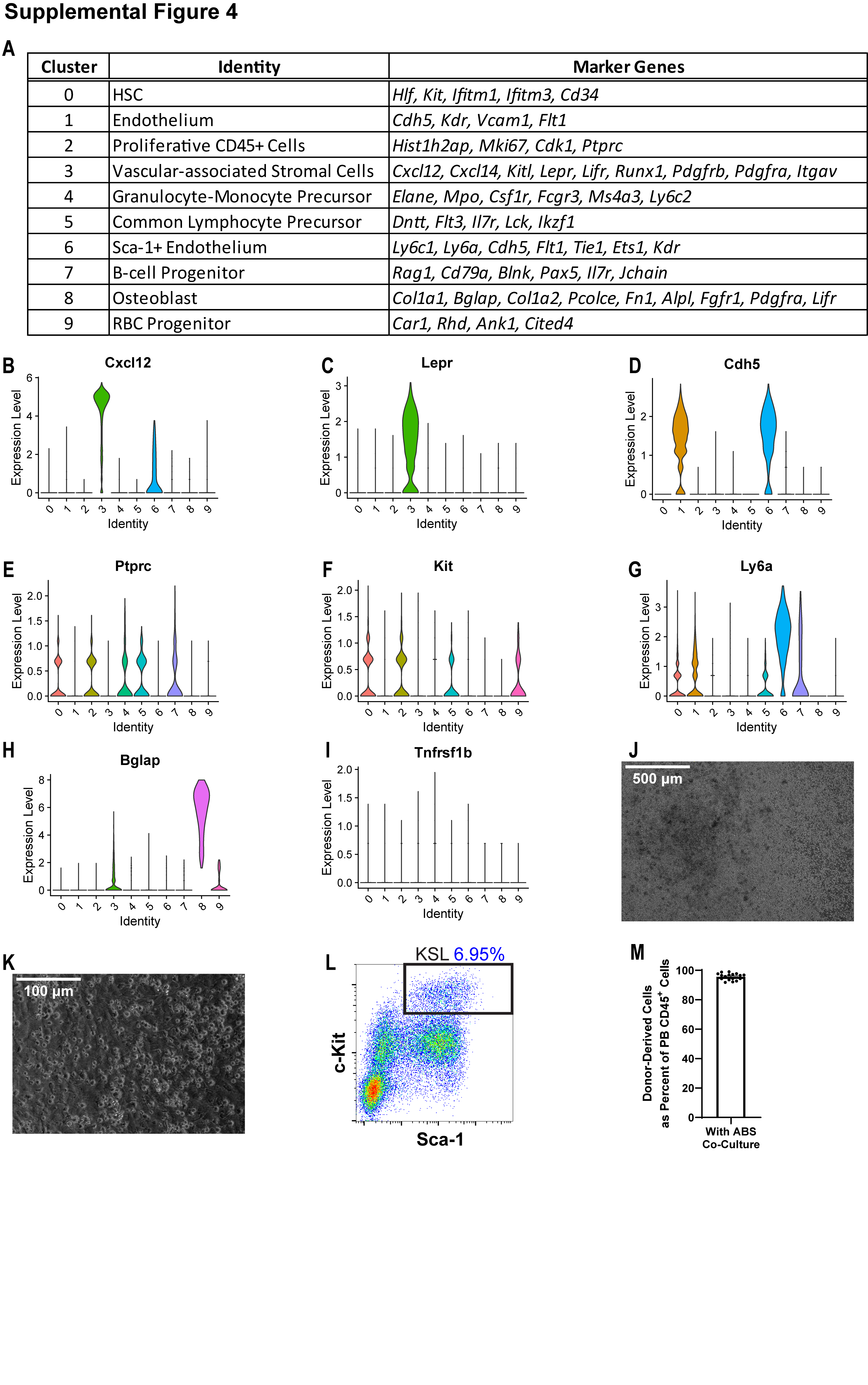

Supplement: S4 Fig — (A–I) From reanalyzed scRNA-seq data of Tikhonova and colleagues of BM niche cell types [49], table with cluster assignments and marker genes (A), violin plot of expression of Cxcl12 (B), Lepr (C), Cdh5 (D), Ptprc (E), Kit (F), Ly6a (G), Bglap (H), and Tnfrsf1b (I). (J–L) Hematopoietic and stromal cocultures after 7 days, representative bright-field image of coculture at 4× magnification (J), representative bright-field image of coculture at 20× magnification (K), representative flow cytometric plot of Live/CD45+ cells with KSL cells as a percentage of Lineage− cells (L). (M) Percent of donor-derived PB CD45+ 1 month after transplantation of hematopoietic and stromal coculture cells into 9.5 Gy irradiated mice (n = 17). Processed data for this figure can be found in S1 Data. The raw flow cytometry data, gating schema, and staining profile relevant to S4L Fig are deposited on Flow Repository under accession number FR-FCM-Z628. BM, bone marrow; KSL, Kit+/Sca-1+/Lineage−; PB, peripheral blood; scRNA-seq, single-cell RNA-sequencing. (TIF) [file pbio.3001746.s004.tif]

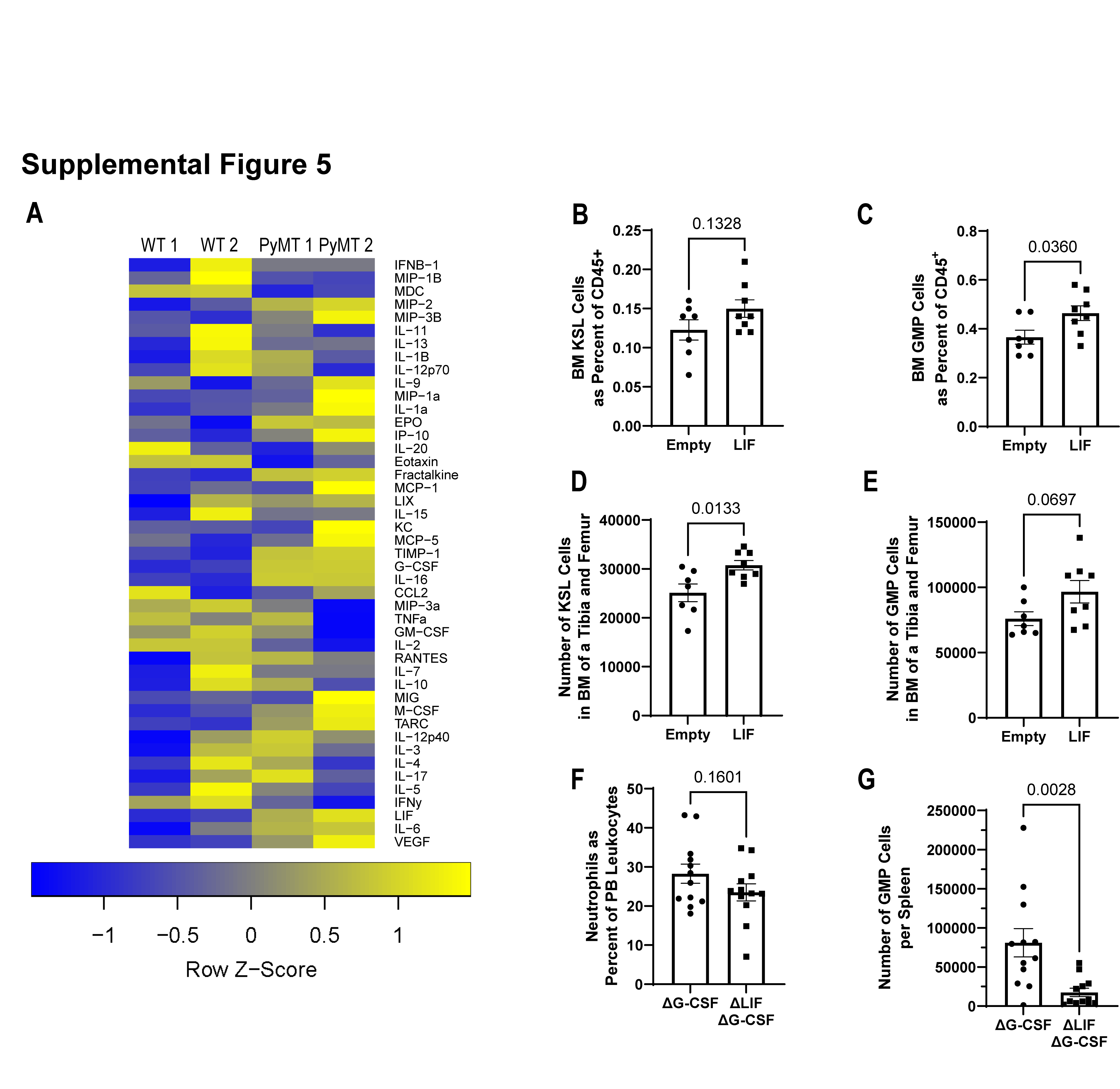

Supplement: S5 Fig — (A) Heatmap showing z-scores of log-normalized expression of 44 cytokines from the serum of MMTV-PyMT+, tumor-bearing animals (PyMT), or age-matched, nontumor-bearing littermates (WT) (n = 2). (B–E) In mice with 10 days of LIF overexpression or empty vector control, KSL cells as a fraction of total BM CD45+ cells (B, n = 7–8), GMP cells as a fraction of total BM CD45+ cells (C, n = 7–8), KSL cells per leg (D, n = 7–8), GMP cells per leg (E, n = 7–8). (F, G) Twenty-eight days after subcutaneous injection of 2.5 × 10^5^ PyMT-B6 ΔG-CSF parental cells or ΔG-CSFΔLIF cells, PMNs in the PB as a percent of total leukocytes (F, n = 12), GMP cells per spleen (G, n = 12). Processed data for this figure can be found in S1 Data. The raw cytokine profiling results used to generate S5A Fig are available as S2 Data file. BM, bone marrow; GMP, granulocyte–monocyte precursor; KSL, Kit+/Sca-1+/Lineage−; LIF, leukemia inhibitory factor; MMTV, murine mammary tumor virus; PB, peripheral blood; PMN, polymorphonuclear neutrophil; PyMT, polyomavirus middle T antigen; WT, wild type. (TIF) [file pbio.3001746.s005.tif]

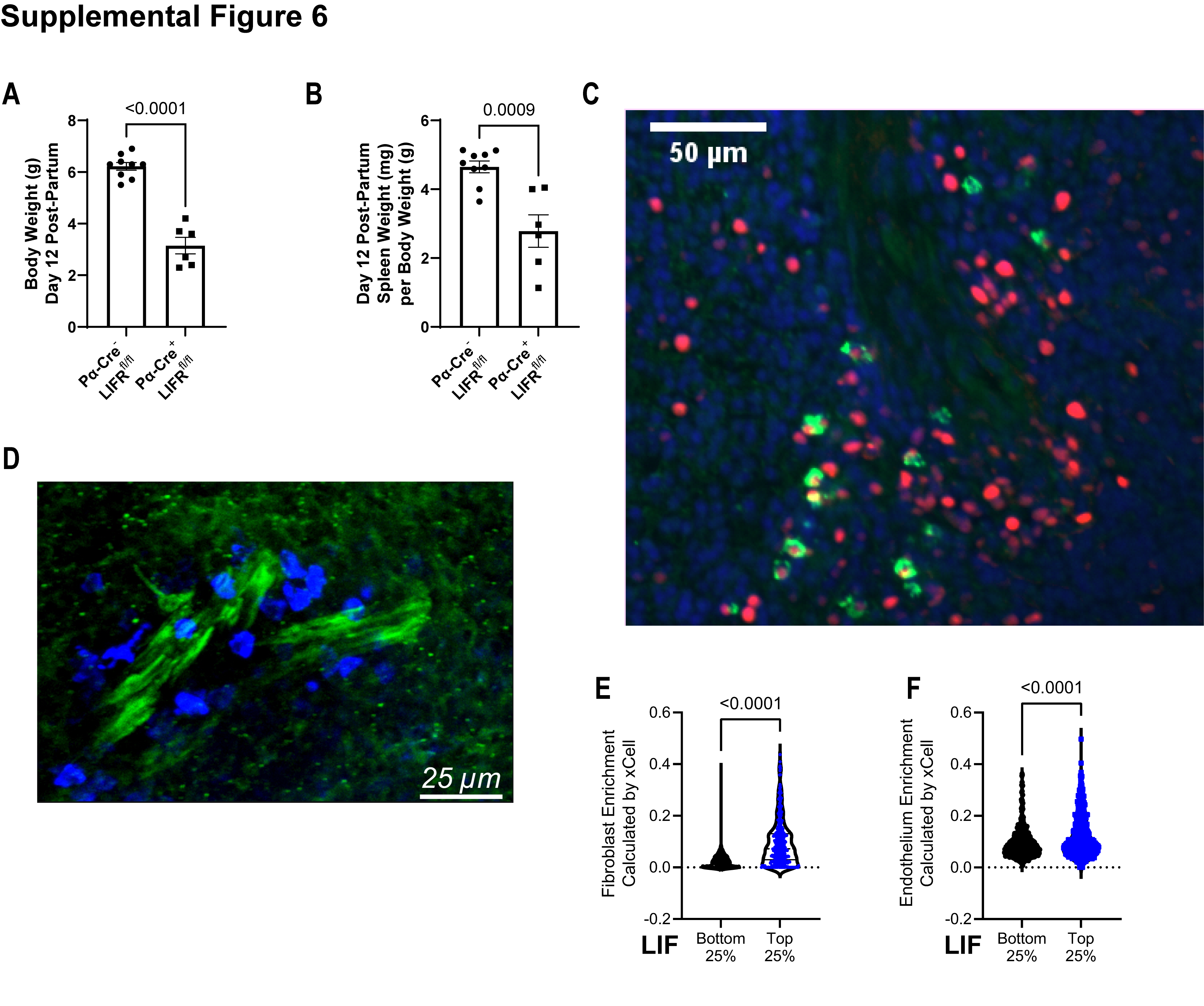

Supplement: S6 Fig — (A, B) In day 12 postpartum LIFRflox mice with PDGFRα-Cre+ or PDGFRα-Cre− littermates, body weight (A, n = 6–9, contains male mice), splenic weight as a fraction of total body weight (B, n = 6–9, contains male mice). (C) Representative immunofluorescence image of the spleen after LIF overexpression with PDGFRα+ cells in green, Ki67+ nuclei in red, and DAPI+ nuclei in blue. (D) Representative confocal image of spleen after LIF overexpression with PDGFRα+ cells in green and c-Kit+ cells in blue. (E, F) Enrichment of fibroblasts (E) and endothelial cells (F) as calculated by xCell from RNA-seq data of human tumors split by top and bottom quartile of LIF expression (n = 416–417). Processed data for this figure can be found in S1 Data. LIF, leukemia inhibitory factor. (TIF) [file pbio.3001746.s006.tif]

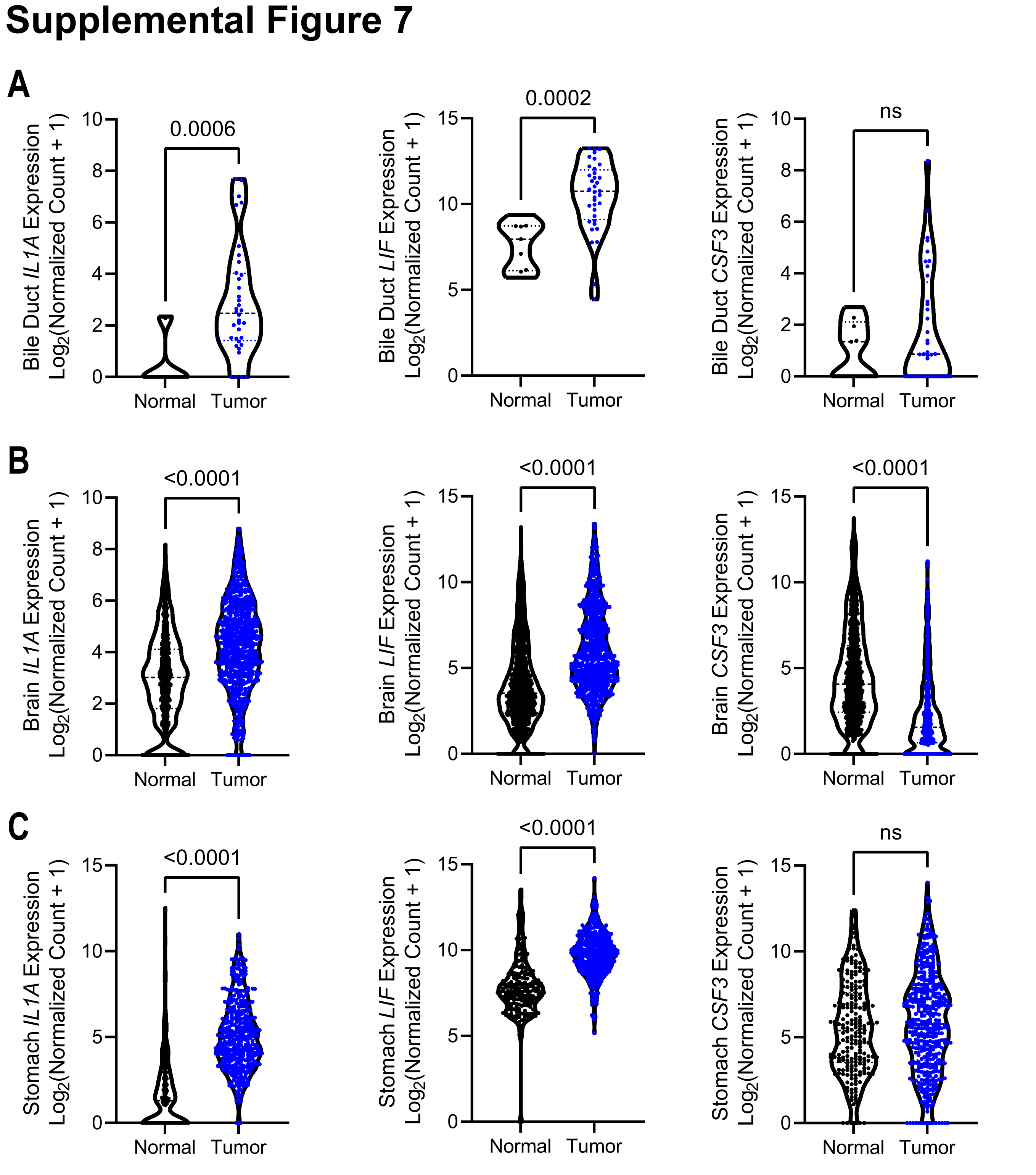

Supplement: S7 Fig — (A–C) RNA-seq expression of IL1A, LIF, and CSF3 expression in tumor compared to normal tissue for bile duct (A, n = 9–36), brain (B, n = 689–1,146), and gastric (C, n = 210–414) tumors. Processed data for this figure can be found in S1 Data. LIF, leukemia inhibitory factor; RNA-seq, RNA-sequencing. (TIF) [file pbio.3001746.s007.tif]
